# The role of oxidative balance score in Cardiovascular-Kidney-Metabolic syndrome progression and mortality: insights from NHANES 1999–2020

**DOI:** 10.3389/fnut.2025.1597693

**Published:** 2025-07-01

**Authors:** Diya Xie, Fangqin You, Lihang Yang, Cheng Li, Fengmin Liu

**Affiliations:** ^1^Department of General Surgery, Fuzhou First General Hospital Affiliated with Fujian Medical University, Fuzhou, Fujian, China; ^2^Department of Endocrinology, Fuzhou First General Hospital Affiliated with Fujian Medical University, Fuzhou, Fujian, China

**Keywords:** oxidative balance score, Cardiovascular-Kidney-Metabolic syndrome, NHANES, mediation analysis, mortality

## Abstract

**Aims:**

Cardiovascular-Kidney-Metabolic Syndrome (CKM) is a significant public health issue. This research explored the link between oxidative balance score (OBS) and the advancement of CKM, and assessed mortality risks across CKM stages in a U. S. cohort.

**Methods:**

Data from 10 NHANES cycles (1999–2020) were analyzed, including 19,433 participants for primary analysis and 16,467 for survival analysis. Multinomial regression, Cox models, survival analysis and mediation analysis were employed to evaluate the relationships.

**Results:**

The OBS was robustly associated with CKM stages, with each increment reducing the odds of CKM stages 1–4 (OR 0.93–0.90, all *p* < 0.001). The highest OBS quartile (Q4) lowered advanced CKM odds (OR 0.77, 95% CI 0.59–0.99, *p* = 0.045). Higher OBS values were associated with a lower risk of all-cause mortality (HR 0.97, 95%CI 0.95–0.99, *p* < 0.001) and cardiovascular disease (CVD) mortality (HR 0.96, 95% CI 0.93–0.99, *p* = 0.012) in individuals with non-advanced chronic kidney disease (CKD), and with a lower risk of CVD mortality (HR 0.97, 95% CI 0.94–0.99, *p* = 0.014) in those with advanced CKD. Kaplan–Meier curves showed better survival in higher OBS quartiles, especially for non-advanced CKM. Inflammatory markers (Ln-WBC and Ln-SUA) mediated 26.08 and 15.17% of the total effect in advanced CKM.

**Conclusion:**

Improving oxidative balance may mitigate CKM progression and mortality risks. Additional studies are required to clarify the mechanisms and public health significance of OBS in CKM.

## 1 Introduction

Cardiovascular-Kidney-Metabolic (CKM) syndrome is defined as a health disorder that is a systemic disease caused by the pathophysiological interactions among obesity, type 2 diabetes (T2DM), chronic kidney disease (CKD), and cardiovascular disease (CVD) (1). This syndrome reflects the interplay among metabolic risk factors, chronic kidney disease, and the cardiovascular system, and has a profound impact on morbidity and mortality (2). Current treatment strategies often fall short due to the complex interrelationships among these conditions (3). Understanding the underlying mechanisms and identifying modifiable risk factors are fundamental to the control and treatment strategies for CKM syndrome.

The connection between oxidative stress and CKM syndrome has increasingly drawn attention. Oxidative stress is now acknowledged as a fundamental contributor to the mechanism of CKM syndrome (4). Upregulated reactive oxygen species (ROS) levels and impaired antioxidant systems are implicated in the emergence and deterioration of metabolic disorders, CKD, and CVD (5). The balance between antioxidants and pro-oxidants, known as oxidative balance, may significantly influence CKM syndrome risk and associated mortality (6). Prior researches have shown that higher dietary antioxidant intake and healthier lifestyle factors are linked to reduced oxidative stress and improved health outcomes (7, 8). For instance, curcumin, a naturally occurring antioxidant, has been shown to have significant bioaccessibility and cellular uptake when formulated in submicron lipid carriers, offering potential therapeutic benefits (9). However, the comprehensive impact of oxidative balance, encompassing both dietary and lifestyle factors, on CKM syndrome and mortality remains underexplored.

The oxidative balance score (OBS) serves as a holistic indicator that consolidates a wide range of dietary nutrients and lifestyle factors into a single measure. This comprehensive approach allows for a detailed assessment of an individual’s overall oxidative status, providing valuable insights into the balance between pro-oxidant and antioxidant factors within the body (10). This score provides a holistic view of antioxidant exposure and its potential influence on health outcomes. Recent studies have demonstrated the utility of OBS in predicting various chronic diseases, including cancer and cardiovascular diseases (11, 12). The association between OBS and CKM syndrome, along with mortality outcomes across different CKM stages, has not been thoroughly explored in large-scale, nationally representative populations.

This research utilizes the National Health and Nutrition Examination Survey (NHANES) database, employing a diverse array of statistical techniques, including regression models, Cox proportional hazards models, and survival analysis. The main goals are to examine the associations between the OBS and the stages of CKM syndrome, as well as to evaluate the corresponding mortality rates. Furthermore, the study investigates the potential mediating effects of inflammatory markers on these relationships. The results underscore the importance of oxidative balance in CKM syndrome, a condition that is increasingly acknowledged as a significant driver of morbidity and mortality, thereby highlighting its relevance to public health.

## 2 Methods

### 2.1 Study design and participants

**Figure 1:**
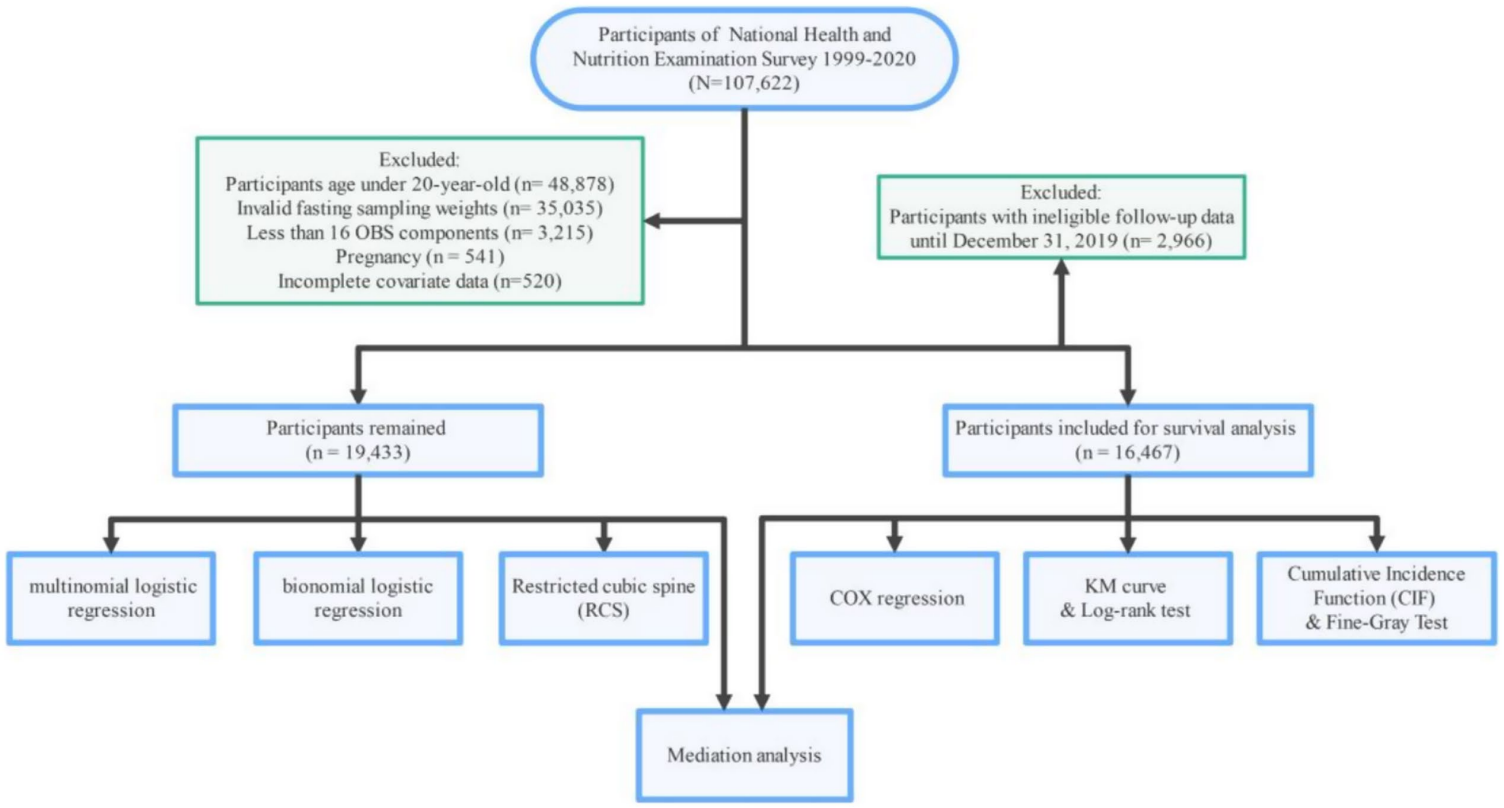
Flowchart of the study, National Health and Nutrition Examination Survey (NHANES), USA, 1999–2020.

The NHANES initiative employs a rigorous sampling approach to assemble a cohort that is representative of the whole U. S. collective, conducting comprehensive health and nutrition evaluations every 2 years. The study protocol received approval from the NCHS Ethics Review Board, and all participants granted written consent prior to their involvement. This analysis encompasses data from 107,622 individuals, spanning 10 NHANES cycles from 1999 to 2020. The exclusion criteria were applied in the following manner: (1) participants aged under 20 years (*n* = 48,878); (2) records with invalid fasting sampling weight (*n* = 35,035); (3) individuals with less than 16 observed OBS components (*n* = 3,215); and (4) participants who were pregnant (*n* = 541) and those with incomplete covariate data (*n* = 520). A final total of 19,433 participants were included in the primary analysis. For the survival analysis, participants with ineligible follow-up data were excluded, resulting in a sample size of 16,467. Figure 1 provides a comprehensive visual representation of the participant selection process, outlining each stage of recruitment and the criteria applied at each step to ensure the final cohort was well-defined and representative of the target population.

### 2.2 CKM definitions and staging

The identification of CKM syndrome in this study was guided by the criteria established by the American Heart Association (AHA), which captures the interplay between metabolic risk factors, CVD and CKD (3). The stages of CKM syndrome were categorized as follows: Stage 0 (no CKM risk factors present), Stage 1 (characterized by excess or dysfunctional adiposity), Stage 2 (presence of metabolic risk factors such as hypertension, diabetes, or hyperlipidemia, or CKD classified as moderate-to-high risk according to Kidney Disease Improving Global Outcomes (KDIGO) criteria (13)), Stage 3 (very high-risk CKD or a high predicted 10-year CVD risk assessed using REVENT equations (14)) and Stage 4 (clinical manifestation of cardiovascular disease). For analysis, participants were divided into two main groups: non-advanced CKM (stages 0, 1, and 2) and advanced CKM (stages 3 and 4). They were further classified into non-severe CKM (stages 0, 1, 2, and 3) and severe CKM (stage 4). A detailed description of the CKM syndrome criteria is provided in Supplementary Table S1.

### 2.3 Oxidative balance score calculation

The OBS was calculated based on 20 components, which included 16 dietary nutrients and 4 lifestyle factors. These components were categorized into 15 antioxidants and 5 pro-oxidants. The dietary nutrients covered a range of essential elements such as dietary fiber, carotenoids (measured in retinol equivalents), riboflavin, niacin, vitamin B6, total folate, vitamin B12, vitamin C, vitamin E (expressed as alpha-tocopherol equivalents), calcium, magnesium, zinc, copper, selenium, total fat, and iron. Dietary intake was assessed using the USDA’s Automated Multiple-Pass Method (AMPM), which involves trained interviewers conducting two 24 h dietary recalls for each participant. The first recall was collected in-person at the Mobile Examination Center (MEC), while the second recall was obtained via telephone 3–10 days later. The collected data were then linked to the USDA’s Food and Nutrient Database for Dietary Studies (FNDDS) to determine the nutrient intake values (15). The lifestyle factors assessed were physical activity levels, body mass index (BMI), alcohol consumption, and smoking status (assessed via serum cotinine levels).

For the dietary constituents, carotenoids were measured as carotenoids RE in the 1999–2000 cycle, while in other cycles, *β*-cryptoxanthin, alpha-and beta-carotene were combined and converted. The lifestyle factor of physical activity (PA) was quantified in metabolic equivalent of task (MET) minutes per week. Given that questionnaires for PA changed around 2007, and the scale for carotenoids RE also varied before and after 2000, we performed weighted quantile for these components separately by cycles to minimize potential biases.

Regarding BMI, considering that CKM definitions stratify BMI differently for Asian and other populations, we adjusted the OBS BMI thresholds accordingly. Scores for remaining components were assigned based on gender-stratified tertiles: antioxidants assigned 0, 1, and 2 points from the lowest to the highest tertiles, while pro-oxidants received 2, 1, and 0 points. For missing values, antioxidants were scored as 0 and pro-oxidants as 2. For example, missing PA was scored as 0, and missing cotinine was scored as 2 (16). The total OBS was derived by aggregating the individual scores, spanning a range from 3 to 36. Elevated OBS values correspond to higher levels of antioxidant exposure, with specific scoring details for each component outlined in Supplementary Table S2.

### 2.4 Mortality outcomes

Mortality data were sourced from the National Death Index (NDI), maintained by the Centers for Disease Control and Prevention (CDC) (17). The follow-up duration extended from the date of the initial interview to December 31, 2019. Cardiovascular mortality was determined using ICD-10 codes I00-I09, I11, I13, and I20-I51, whereas all-cause mortality encompassed deaths from any cause.

### 2.5 Covariates

Adjustment variables included age, sex (female, male), race (Mexican American, Non-Hispanic White, Non-Hispanic Black, Other), poverty-income ratio (PIR; categorized as ≤1.3, 1.3–3.5, >3.5), level of education (Less than High School, High School, More than High School), and marital situation (Never Married, Married/Living with Partner, Other). Additionally, white blood cell count, serum uric acid, and dietary intakes of energy, protein, carbohydrate, sodium, and potassium were included, as these factors may be associated with CKM components (18).

### 2.6 Statistical analysis

#### 2.6.1 Descriptive statistics

The baseline characteristics of the study population were summarized using statistical methods. Continuous variables were presented as medians along with interquartile ranges (IQR), while categorical variables were shown as frequencies and percentages. The distribution of CKM stages across different NHANES cycles and OBS quartiles was visually assessed using figures and tables. Complex survey design and fasting sampling weights (WTSAF4YR in 1999–2012, WTSAF2YR in 2003–2016 and WTSAFPRP in 2017–2020) were incorporated to account for the stratified, multistage probability sampling of NHANES, ensuring that results were representative of the US demographic (19).

#### 2.6.2 Regression models

To evaluate the link between OBS and CKM stages, both unadjusted (Model 1) and fully adjusted (Model 2) regression models were applied. The fully adjusted model included covariates such as age, gender, race, PIR, education, marital status, and log-transformed values of white blood cell count, serum uric acid, and dietary intakes (energy, protein, carbohydrate, sodium, and potassium). OBS was divided into quartiles (Q1–Q4) to assess the dose–response relationship. Odds ratios (OR) and 95% confidence intervals (CI) were calculated using multinomial logistic regression, comparing each CKM stage to stage 0.

For individuals with advanced CKM (stages 3 and 4) and severe CKM (stage 4), binary logistic regression analyses were conducted. Furthermore, Cox models were used to assess the links between OBS and mortality (all-cause and CVD) across different CKM stages. The models were stratified by CKM stages (each of the five stages, non-advanced and advanced stages).

#### 2.6.3 Kaplan–Meier curves and log-rank test

Kaplan–Meier curves were generated to show survival differences by OBS quartiles for all-cause and CVD mortality in participants with advanced and non-advanced CKM, with log-rank tests used for comparisons.

#### 2.6.4 Cumulative Incidence Function and Fine-Gray test

To account for competing risks in the analysis of cardiovascular mortality, CIF (20) and Fine-Gray tests (21) were employed. The CIF was used to quantify the total occurrence of cardiovascular mortality, and the Fine-Gray test was used to compare the CIFs across different OBS quartiles.

#### 2.6.5 Mediation analysis

Mediation analysis was conducted to evaluate the extent to which inflammatory markers (Ln-WBC and Ln-SUA) mediate the association between OBS and CKM outcomes. Logistic regression models were used for CKM stage, and parametric accelerated failure time models (22) were used for mortality. Effects from survival models should be interpreted as impacts on survival time, not mortality rates. We used 1,000 bootstrap iterations to estimate mediation effects robustly.

Statistical assessments were completed using R version 4.4.2, with the following packages: *survey, rms, survival, jskm, cmprsk, mediation* (23) and *ggplot2*. A two-sided *p*-value <0.05 was considered statistically significant.

## 3 Results

### 3.1 Baseline characteristics

**Figure 2:**
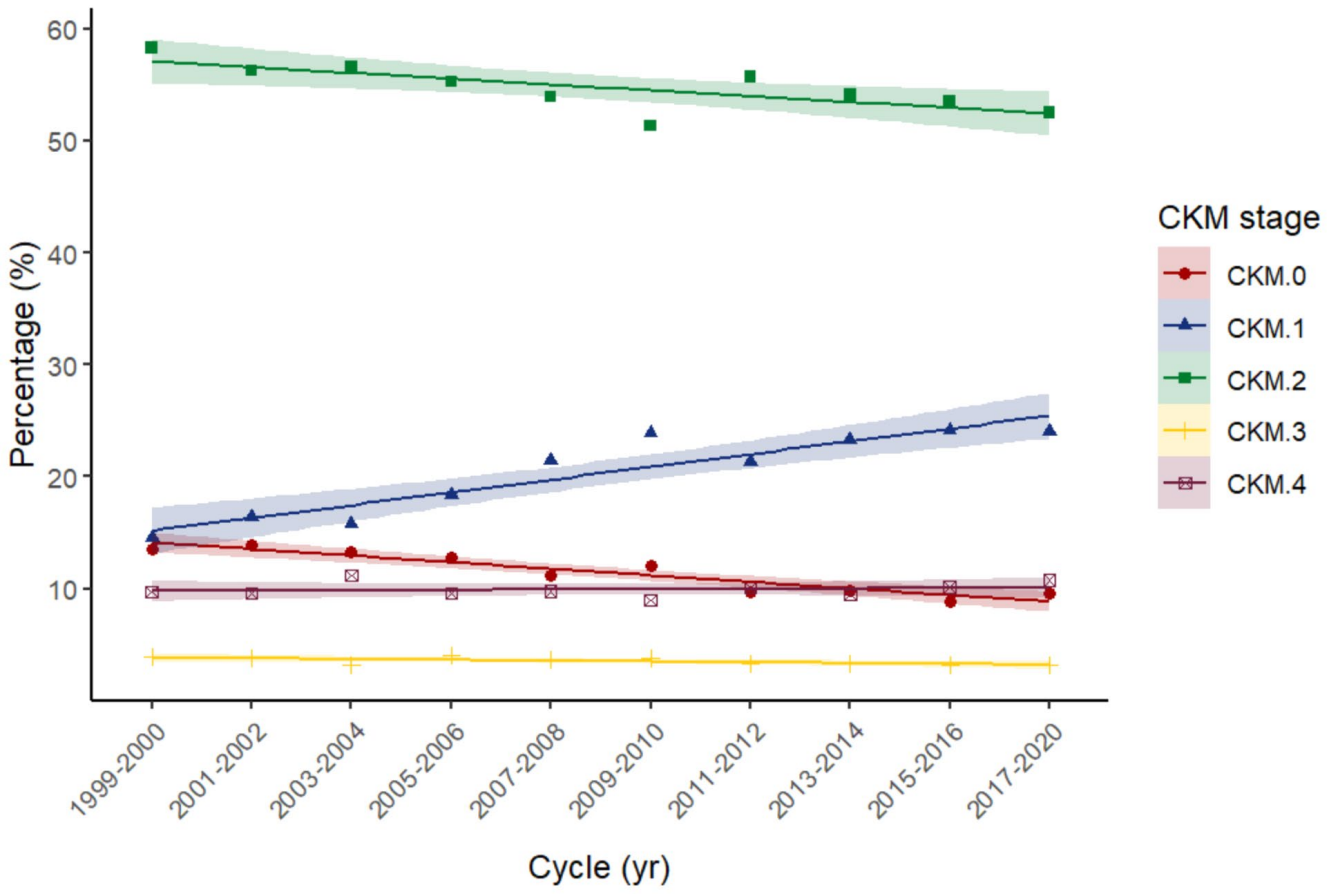
Trend of each Cardiovascular-Kidney-Metabolic syndrome (CKM) stage across NHANES cycles 1999–2020. Linear fits applied to illustrate the trend over time.

This work included 19,433 participants (185,175,890 of weighted population), comprising 9,957 males and 9,476 females. The distribution of CKM stages in our study across different NHANES cycles reveals distinct trends over time (Figure 2; Supplementary Table S3). CKM stage 2 remains the most prevalent stage throughout the study period, although its proportion has shown a slight decline from 58.34% in the 1999–2000 cycle to 52.48% in the 2017–2020 cycle. CKM stage 0 has experienced a notable decrease in prevalence, declining from 13.56% in 1999–2000 to 9.59% in 2017–2020. Conversely, CKM stage 1 has shown a consistent increase, rising from 14.50% in 1999–2000 to 23.99% in 2017–2020. The remaining stages (stages 3 and 4) have exhibited fluctuations in prevalence but have not shown clear monotonic trends.

The baseline characteristics stratified by CKM stages (Table 1) reveal notable differences across key demographic and health indicators. CKM stage 4, representing the most advanced stage, is characterized by a significantly higher median age compared to earlier stages. This stage also has a higher proportion of females. Education levels show a clear gradient, with CKM stage 0 having the highest proportion of individuals with education above high school, while CKM stage 4 has the lowest. In terms of poverty income ratio (PIR), CKM stage 3 and 4 has a higher proportion of individuals with lower income levels (PIR ≤ 1.3). Dietary intake patterns also vary across CKM stages, with stage 0 showing the highest energy and protein intake, while stage 4 has the lowest. Additionally, CKM stage 4 has the highest levels of white blood cells and serum uric acid. These findings indicate that advanced CKM stages are associated with older age, lower educational attainment, higher poverty levels, reduced dietary intake, and increased inflammation and metabolic burden. Notably, the total OBS score decreases progressively from CKM stage 0 to stage 4.

**Table 1:** Baseline characteristics of participants according to the CKM stages, NHANES 1999–2020.

Characteristic	Overall	CKM 0	CKM 1	CKM 2	CKM 3	CKM 4	*p*-value^b^
	*n* = 19,433	*n* = 1,676 (11%)	*n* = 3,604 (21%)	*n* = 10,537 (55%)	*n* = 1,171 (3.5%)	*n* = 2,445 (10.0%)	
	*N* = 185,175,890^a^	*N* = 20,955,041	*N* = 38,258,211	*N* = 101,038,179	*N* = 6,453,776	*N* = 18,470,683	
Age (years old)	47.00 (34.00, 60.00)	33.00 (25.00, 43.00)	38.00 (28.00, 50.00)	48.00 (36.00, 59.00)	79.00 (74.00, 80.00)	66.00 (56.00, 75.00)	**<0.001**
Gender							**<0.001**
Female	9,476 (48.18%)	608 (33.94%)	1,625 (46.63%)	5,166 (50.30%)	711 (54.97%)	1,366 (53.60%)	
Male	9,957 (51.82%)	1,068 (66.06%)	1,979 (53.37%)	5,371 (49.70%)	460 (45.03%)	1,079 (46.40%)	
Poverty income ratio (PIR)							**<0.001**
≤1.3	5,162 (18.76%)	394 (16.05%)	890 (17.48%)	2,780 (18.58%)	334 (24.22%)	764 (23.56%)	
1.3–3.5	6,898 (34.08%)	541 (29.34%)	1,236 (33.39%)	3,669 (33.78%)	502 (44.22%)	950 (39.04%)	
≤3.5	5,787 (40.63%)	618 (48.05%)	1,201 (43.21%)	3,211 (41.15%)	230 (23.60%)	527 (30.04%)	
Race							**<0.001**
Mexican American	3,314 (7.74%)	228 (5.97%)	728 (10.33%)	1,953 (8.13%)	144 (4.09%)	261 (3.54%)	
Non-Hispanic White	3,863 (10.74%)	251 (7.60%)	720 (11.44%)	2,174 (11.02%)	206 (10.83%)	512 (11.28%)	
Non-Hispanic Black	8,994 (69.72%)	881 (75.09%)	1,439 (65.04%)	4,563 (68.68%)	710 (77.52%)	1,401 (76.29%)	
Other race	3,262 (11.79%)	316 (11.34%)	717 (13.19%)	1,847 (12.17%)	111 (7.56%)	271 (8.89%)	
Education							**<0.001**
Below high school	2,122 (5.46%)	81 (2.36%)	287 (4.47%)	1,124 (5.22%)	237 (13.37%)	393 (9.60%)	
High school	7,162 (34.70%)	489 (26.74%)	1,180 (30.67%)	3,974 (35.59%)	474 (43.33%)	1,045 (44.22%)	
Higher than high school	10,149 (59.83%)	1,106 (70.90%)	2,137 (64.86%)	5,439 (59.18%)	460 (43.30%)	1,007 (46.18%)	
Marital status							**<0.001**
Never married	3,206 (16.81%)	584 (31.16%)	835 (21.90%)	1,601 (14.91%)	38 (2.91%)	148 (5.26%)	
Married/living with partner	11,987 (65.20%)	933 (59.55%)	2,268 (65.10%)	6,709 (67.13%)	657 (56.82%)	1,420 (64.20%)	
Others	4,240 (17.99%)	159 (9.29%)	501 (12.99%)	2,227 (17.96%)	476 (40.27%)	877 (30.53%)	
Energy intake (Kcal/d)	1,998.10 (1,527.00, 2,571.88)	2,058.50 (1,562.47, 2,602.01)	2,066.50 (1,596.87, 2,623.07)	2,031.00 (1,540.95, 2,627.50)	1,661.04 (1,277.98, 2,095.17)	1,771.00 (1,357.46, 2,254.00)	**<0.001**
Protein intake (g/d)	76.62 (57.24, 100.66)	75.18 (57.90, 100.13)	79.88 (60.75, 103.20)	78.07 (57.77, 102.46)	64.98 (48.43, 79.97)	68.66 (51.72, 90.90)	**<0.001**
Carbohydrate intake (g/d)	237.51 (178.19, 310.85)	248.60 (184.89, 329.60)	244.46 (183.82, 317.04)	239.14 (180.26, 314.16)	202.09 (154.08, 262.74)	214.84 (159.96, 276.15)	**<0.001**
Sodium intake (mg/d)	3,227.67 (2,401.00, 4,242.50)	3,231.09 (2,425.07, 4,226.31)	3,350.32 (2,539.59, 4,337.19)	3,286.50 (2,419.96, 4,317.59)	2,694.53 (2,014.30, 3,485.89)	2,908.79 (2,114.59, 3,918.50)	**<0.001**
Potassium intake (mg/d)	2,552.50 (1,919.51, 3,255.00)	2,561.02 (1,930.94, 3,251.13)	2,567.00 (1,933.20, 3,305.43)	2,570.50 (1,939.82, 3,284.00)	2,373.46 (1,827.62, 2,987.41)	2,434.88 (1,819.00, 3,163.45)	**<0.001**
White blood cells (10^9^/L)	6.40 (5.40, 7.80)	5.90 (4.90, 7.00)	6.10 (5.20, 7.40)	6.60 (5.60, 8.00)	6.90 (5.80, 8.20)	6.90 (5.70, 8.20)	**<0.001**
Serum uric acid (umol/L)	321.20 (267.70, 380.70)	261.70 (226.00, 315.20)	297.40 (255.80, 350.90)	333.10 (279.60, 386.60)	345.00 (297.40, 410.40)	345.00 (285.50, 410.40)	**<0.001**
Total OBS	20.00 (14.00, 26.00)	23.00 (17.00, 28.00)	21.00 (15.00, 26.00)	20.00 (14.00, 25.00)	17.00 (13.00, 23.00)	18.00 (12.00, 24.00)	**<0.001**
Diet OBS	16.00 (10.00, 22.00)	18.00 (12.00, 23.00)	17.00 (11.00, 22.00)	16.00 (10.00, 22.00)	13.00 (8.00, 19.00)	14.00 (8.00, 19.24)	**<0.001**
Life OBS	4.00 (3.00, 5.00)	5.00 (4.00, 6.00)	4.00 (3.00, 5.00)	4.00 (3.00, 5.00)	4.00 (3.00, 5.00)	4.00 (3.00, 5.00)	**<0.001**
Body mass index (kg/m^2^)	27.79 (24.10, 32.22)	22.10 (20.51, 23.49)	27.40 (25.20, 30.70)	29.24 (25.68, 33.81)	27.69 (24.62, 31.00)	29.10 (25.40, 33.80)	**<0.001**
Waist (cm)	97.30 (87.00, 108.40)	79.30 (74.50, 83.60)	94.50 (88.00, 103.00)	101.20 (91.80, 111.60)	102.00 (93.71, 110.30)	103.70 (94.20, 115.02)	**<0.001**
Glucose (mg/dL)	99.00 (92.00, 107.50)	90.00 (86.00, 94.72)	97.00 (91.00, 103.00)	101.00 (94.00, 110.00)	111.00 (99.00, 132.24)	107.00 (96.90, 122.00)	**<0.001**
Glycohemoglobin (%)	5.40 (5.10, 5.70)	5.10 (4.92, 5.30)	5.30 (5.10, 5.50)	5.50 (5.20, 5.80)	5.90 (5.50, 6.50)	5.70 (5.40, 6.20)	**<0.001**
Triglycerides (mg/dL)	105.00 (72.00, 155.00)	68.00 (52.00, 88.00)	80.00 (60.00, 102.00)	135.00 (89.00, 185.00)	124.00 (86.00, 174.00)	121.91 (85.00, 180.00)	**<0.001**
HDL (mg/dL)	51.00 (42.00, 62.00)	61.00 (51.51, 72.00)	55.00 (47.00, 64.00)	48.00 (40.00, 59.00)	50.00 (42.00, 59.00)	48.00 (40.00, 59.00)	**<0.001**
Hypertension	10,562 (49.35%)	–	–	7,513 (70.34%)	1,043 (89.69%)	2,006 (78.60%)	**<0.001**
Metabolic syndromes	8,010 (38.18%)	–	–	5,958 (55.94%)	634 (55.69%)	1,418 (57.33%)	**<0.001**
Pre-diabetes	9,570 (46.24%)	–	1,681 (45.23%)	5,794 (53.34%)	668 (57.18%)	1,427 (58.01%)	**<0.001**
Glycemic status							**<0.001**
Diabetes	3,706 (14.42%)	–	–	2,159 (16.67%)	591 (51.45%)	956 (35.35%)	
Impaired fasting glucose (IFG)	1,862 (9.24%)	–	253 (7.26%)	1,183 (11.02%)	123 (10.40%)	303 (13.64%)	
Impaired Glucose Tolerance (IGT)	1,132 (5.25%)	34 (1.65%)	152 (3.78%)	732 (6.46%)	79 (6.89%)	135 (5.22%)	
Normoglycemia	12,731 (71.09%)	1,642 (98.35%)	3,198 (88.96%)	6,462 (65.85%)	378 (31.26%)	1,051 (45.79%)	
CKD prognosis	3,338 (12.83%)	–	–	1,675 (13.59%)	650 (54.65%)	1,013 (35.14%)	**<0.001**
High CVD 10 years risk	3.57 (1.32, 9.09)	0.90 (0.46, 1.72)	1.43 (0.68, 3.09)	3.93 (1.73, 8.16)	24.62 (21.70, 28.92)	12.25 (6.45, 20.83)	**<0.001**
ASCVD	2,050 (8.48%)	–	–	–	–	2,050 (85.04%)	**<0.001**
Congestive heart failure	639 (2.43%)	–	–	–	–	639 (24.54%)	**<0.001**
NHANES cycles							**<0.001**
1999–2000	1,518.00 (7.65%)	154.00 (9.17%)	222.00 (5.37%)	845.00 (8.18%)	113.00 (8.55%)	184.00 (7.45%)	
2001–2002	1,977.00 (10.42%)	212.00 (12.85%)	301.00 (8.29%)	1,074.00 (10.76%)	130.00 (11.18%)	260.00 (10.01%)	
2003–2004	1,654.00 (9.11%)	163.00 (10.68%)	228.00 (6.97%)	875.00 (9.45%)	115.00 (8.25%)	273.00 (10.21%)	
2005–2006	1,594.00 (9.13%)	152.00 (10.29%)	277.00 (8.10%)	856.00 (9.25%)	121.00 (10.55%)	188.00 (8.78%)	
2007–2008	1,944.00 (9.14%)	153.00 (9.07%)	368.00 (9.51%)	1,036.00 (9.03%)	125.00 (9.57%)	262.00 (8.92%)	
2009–2010	2,189.00 (9.46%)	204.00 (10.06%)	453.00 (10.94%)	1,166.00 (8.90%)	122.00 (10.24%)	244.00 (8.52%)	
2011–2012	1,911.00 (9.73%)	164.00 (8.35%)	386.00 (10.02%)	1,053.00 (9.93%)	104.00 (9.09%)	204.00 (9.80%)	
2013–2014	1,926.00 (9.58%)	162.00 (8.34%)	408.00 (10.79%)	1,050.00 (9.51%)	93.00 (8.91%)	213.00 (9.15%)	
2015–2016	1,767.00 (9.62%)	113.00 (7.51%)	364.00 (11.27%)	962.00 (9.45%)	99.00 (8.87%)	229.00 (9.79%)	
2017–2020	2,953.00 (16.15%)	199.00 (13.69%)	597.00 (18.74%)	1,620.00 (15.54%)	149.00 (14.80%)	388.00 (17.37%)	

a Median (IQR) for skewed distribution; *n* (unweighted) (weighted percentage%); *N* (weighted).

b Wilcoxon rank-sum test for complex survey samples; chi-squared test with Rao & Scott’s second-order correction.

OBS, oxidative balance score; CKM, Cardiovascular-Kidney-Metabolic syndrome; HDL, high-density lipoprotein; CKD, Chronic Kidney Disease; CVD, Cardiovascular Disease; ASCVD, Atherosclerotic Cardiovascular Disease. Bold values represent statistical significance (*p* < 0.05).

The distribution of baseline characteristics across total OBS quartiles (Supplementary Table S4) also highlights significant variations. Participants in the highest OBS quartile (Q4) exhibited higher education levels and superior metabolic health, characterized by more favorable lipid profiles (lower triglycerides, higher HDL cholesterol), and a lower prevalence of hypertension, diabetes, and related complications compared to those in the lowest quartile (Q1). In contrast, the lowest OBS quartile (Q1) included a higher proportion of individuals with lower incomes (PIR ≤ 1.3) and a greater prevalence of advanced CKM stages (stages 3 and 4). No significant differences in age or gender distribution were noted across OBS quartiles. Dietary intake patterns showed a gradient, with higher energy and protein intake observed in the highest OBS quartile (Q4). Additionally, participants in Q4 had lower levels of white blood cells and serum uric acid. These results indicate a potential association between lower OBS values and more severe CKM stages.

### 3.2 Correlation between OBS and CKM

In fully adjusted multinomial regression models (Table 2), the OBS was inversely associated with the stages of CKM. For total OBS, each score increase was associated with a decreased odds of CKM stages 1 to 4 (compared to stage 0), with odds ratios (OR) of 0.93 (95% CI, 0.93–0.93), 0.91 (95% CI, 0.91–0.91), 0.90 (95% CI, 0.90–0.90), and 0.90 (95% CI, 0.90–0.90), respectively (all *p* < 0.001). For diet OBS, inverse associations were observed with ORs of 0.99, 0.95, 0.92, and 0.94 for CKM stages 1 to 4, respectively (all *p* < 0.001). For life OBS, more pronounced associations were seen with ORs of 0.60, 0.54, 0.61, and 0.51 for CKM stages 1 to 4, respectively (all *p* < 0.001).

**Table 2:** The relationship between OBS and CKM stages using multinomial logistic regression (*n* = 19,433).

OBS type	CKM stages	Model 1			Model 2		
		OR	95% CI	*p*-value	OR	95% CI	*p*-value
Total OBS	0	Ref.			Ref.		
	1	0.97	0.97, 0.97	**<0.001**	0.93	0.93, 0.93	**<0.001**
	2	0.95	0.95, 0.95	**<0.001**	0.91	0.91	**<0.001**
	3	0.92	0.92, 0.92	**<0.001**	0.90	0.90, 0.90	**<0.001**
	4	0.92	0.92, 0.92	**<0.001**	0.90	0.90, 0.90	**<0.001**
Diet OBS	0	Ref.			Ref.		
	1	0.99	0.99, 0.99	**<0.001**	0.99	0.99, 0.99	**<0.001**
	2	0.97	0.97, 0.97	**<0.001**	0.95	0.95, 0.95	**<0.001**
	3	0.93	0.93, 0.93	**<0.001**	0.92	0.92, 0.92	**<0.001**
	4	0.94	0.94, 0.94	**<0.001**	0.94	0.94, 0.94	**<0.001**
Life OBS	0	Ref.			Ref.		
	1	0.60	0.60, 0.60	**<0.001**	0.60	0.60, 0.60	**<0.001**
	2	0.52	0.52, 0.52	**<0.001**	0.54	0.54, 0.54	**<0.001**
	3	0.64	0.64, 0.64	**<0.001**	0.61	0.61	**<0.001**
	4	0.52	0.52, 0.52	**<0.001**	0.51	0.51	**<0.001**

Model 1 Crude model.

Model 2 adjusted for age, gender, race, poverty income ratio (PIR), education level, marital status, log transformed values including white blood cell count, serum uric acid, and dietary intakes (energy, protein, carbohydrate, sodium and potassium).

OBS, oxidative balance score; CKM, Cardiovascular-Kidney-Metabolic syndrome; CI, confidential interval; OR, odds ratio. Bold values represent statistical significance (*p* < 0.05).

In binary logistic regression analyses (Table 3), total OBS was inversely related to advanced CKM (OR 0.98, 95% CI 0.97–1.00, *p* = 0.007) and severe CKM (OR 0.98, 95% CI 0.97–1.00, *p* = 0.01). Life OBS demonstrated a stronger inverse relationship with advanced CKM (OR 0.93, 95% CI 0.89–0.98, *p* = 0.003) and severe CKM (OR 0.90, 95% CI 0.86–0.95, *p* < 0.001). Diet OBS was not significantly associated with advanced CKM (*p* = 0.064) or severe CKM (*p* = 0.167).

**Table 3:** The relationship between OBS and binary CKM staging using binomial logistic regression (*n* = 19,433).

OBS types	Advanced CKM						Severe CKM					
	Model 1			Model 2			Model 1			Model 2		
	OR	95% CI	*p*-value	OR	95% CI	*p*-value	OR	95% CI	*p*-value	OR	95% CI	*p*-value
Total OBS	0.96	0.95, 0.96	**<0.001**	0.98	0.97, 1.0	**0.007**	0.96	0.95, 0.97	**<0.001**	0.98	0.97, 1.00	**0.010**
Total OBS quartile												
Q1	Ref.			Ref.			Ref.			Ref.		
Q2	0.76	0.68, 0.85	**<0.001**	0.92	0.77, 1.09	0.317	0.77	0.67, 0.89	**<0.001**	0.92	0.76, 1.10	0.331
Q3	0.55	0.49, 0.62	**<0.001**	0.79	0.64, 0.97	**0.025**	0.57	0.50, 0.66	**<0.001**	0.8	0.65, 1.0	**0.045**
Q4	0.44	0.38, 0.51	**<0.001**	0.77	0.59, 0.99	**0.045**	0.47	0.39, 0.56	**<0.001**	0.78	0.59, 1.03	0.076
P for trend	0.75	0.72, 0.79	**<0.001**	0.91	0.83, 0.98	**0.019**	0.77	0.73, 0.81	**<0.001**	0.91	0.84, 1.00	**0.043**
Diet OBS	0.96	0.95, 0.96	**<0.001**	0.99	0.97, 1.00	0.064	0.96	0.95, 0.97	**<0.001**	0.99	0.98, 1.00	0.167
Diet OBS quartile												
Q1	Ref.			Ref.			Ref.			Ref.		
Q2	0.75	0.67, 0.83	**<0.001**	0.95	0.80, 1.12	0.525	0.77	0.67, 0.89	**<0.001**	0.97	0.81, 1.16	0.737
Q3	0.54	0.48, 0.61	**<0.001**	0.83	0.67, 1.02	0.076	0.58	0.50, 0.67	**<0.001**	0.87	0.70, 1.08	0.210
Q4	0.43	0.38, 0.50	**<0.001**	0.83	0.64, 1.08	0.165	0.49	0.41, 0.58	**<0.001**	0.9	0.69, 1.16	0.403
P for trend	0.75	0.72, 0.78	**<0.001**	0.93	0.85, 1.01	0.083	0.78	0.74, 0.82	**<0.001**	0.95	0.88, 1.04	0.254
Life OBS	0.95	0.92, 0.98	**0.001**	0.93	0.89, 0.98	**0.003**	0.9	0.87, 0.93	**<0.001**	0.9	0.86, 0.95	**<0.001**
Life OBS quartile												
Q1	Ref.			Ref.			Ref.			Ref.		
Q2	1.03	0.92, 1.16	0.581	0.88	0.76, 1.02	0.098	0.94	0.82, 1.09	0.42	0.85	0.73, 1.00	**0.045**
Q3	0.92	0.80, 1.05	0.214	0.82	0.69, 0.98	**0.033**	0.76	0.65, 0.89	**<0.001**	0.74	0.61, 0.88	**<0.001**
Q4	0.78	0.68, 0.90	**<0.001**	0.8	0.66, 0.96	**0.018**	0.62	0.52, 0.75	**<0.001**	0.69	0.56, 0.85	**<0.001**
P for trend	0.93	0.89, 0.97	**<0.001**	0.92	0.87, 0.98	**0.008**	0.86	0.81, 0.91	**<0.001**	0.88	0.82, 0.94	**<0.001**

Model 1 Crude model.

Model 2 adjusted for age, gender, race, poverty income ratio (PIR), education level, marital status, log transformed values including white blood cell count, serum uric acid, and dietary intakes (energy, protein, carbohydrate, sodium and potassium).

Total OBS: Q1: (0, 14); Q2: (14, 20); Q3: (20, 26); Q4: ≥26; Dietary OBS: Q1: (0, 10); Q2: (10, 16); Q3: (16, 22); Q4: ≥22; Life OBS: Q1: (0, 3); Q2: (3, 4); Q3: (4, 5); Q4: ≥5.

OBS, oxidative balance score; CKM, Cardiovascular-Kidney-Metabolic syndrome; CI, confidential interval; OR, odds ratio. Bold values represent statistical significance (*p* < 0.05).

In quartile analyses, the highest quartile of total OBS (Q4) was linked to reduced odds of advanced CKM (OR 0.77, 95% CI 0.59–0.99, *p* = 0.045), but not severe CKM (*p* = 0.076). The highest quartile of life OBS (Q4) was associated with lower odds of both advanced CKM (OR 0.80, 95% CI 0.66–0.96, *p* = 0.018) and severe CKM (OR 0.69, 95% CI 0.56–0.85, *p* < 0.001). Diet OBS in the highest quartile (Q4) showed no significant association with advanced CKM (*p* = 0.165) or severe CKM (*p* = 0.403).

**Figure 3:**
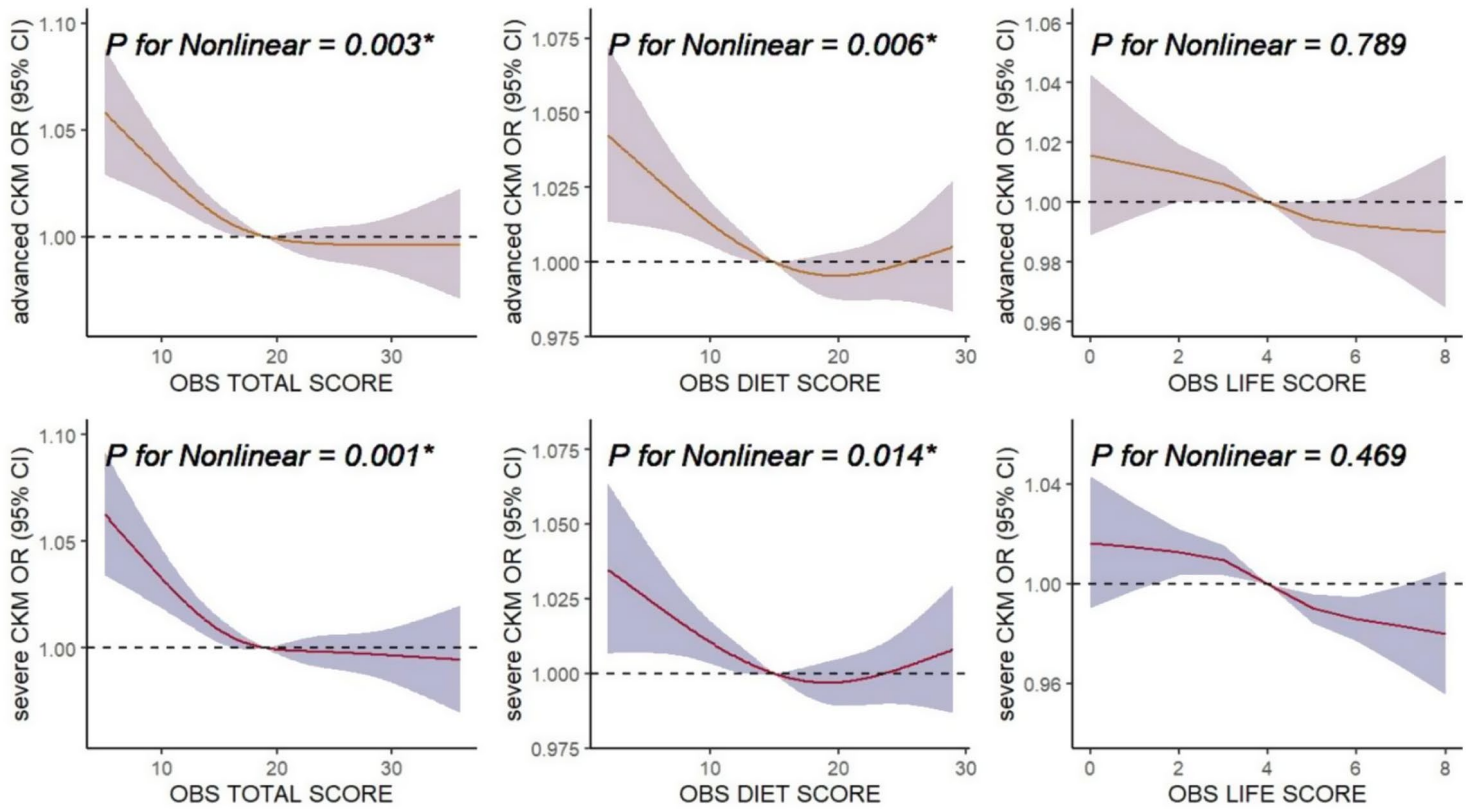
Associations of oxidative balance score (OBS) with advanced CKM and severe CKM. The horizontal dashed line represents to the reference odds ratio of 1.0. All of the models were adjusted for age, gender, race, poverty-income ratio (PIR), education level, marital status, log transformed value of white blood cell count and serum uric acid, as well as dietary intakes of energy, protein, carbohydrate, sodium, and potassium. OR, odds ratio; CI, confidence interval; CKM, Cardiovascular-Kidney-Metabolic syndrome.

Total OBS showed significant non-linear associations with advanced CKM (*p* = 0.003) and severe CKM (*p* = 0.001) in RCS analyses (Figure 3). Diet OBS also exhibited significant non-linear associations with advanced CKM (*p* = 0.006) and severe CKM (*p* = 0.014), with a J-shaped pattern. Life OBS did not show significant non-linear associations with advanced CKM or severe CKM. The results indicate that all three types of OBS (total, diet, and life) are inversely associated with CKM outcomes to varying degrees.

### 3.3 Links between OBS and mortality in different CKM stages

During a median follow-up of 122 days, 2,830 all-cause and 732 CVD mortalities were recorded in the overall analysis. In the non-advanced CKM group, 1,192 all-cause and 254 CVD mortalities were recorded over 128 days; in the advanced CKM group, 1,638 all-cause and 478 CVD mortalities were recorded over 91 days.

For non-advanced CKM (Table 4), higher total OBS was associated with reduced all-cause mortality (HR 0.97, 95% CI 0.95–0.99, *p* < 0.001) and lower CVD mortality (HR 0.96, 95% CI 0.93–0.99, *p* = 0.012). Diet OBS similarly decreased all-cause mortality (HR 0.97, 95% CI 0.96–0.99, *p* = 0.006) and had a notable effect on CVD mortality (HR 0.97, 95% CI 0.94–1.00, *p* = 0.034). Lifestyle OBS was linked to lower all-cause mortality (HR 0.92, 95% CI 0.87–0.97, *p* = 0.003) but not CVD mortality (*p* = 0.20).

**Table 4:** Cox regression of the relationship between OBS and mortality in non-advanced and advanced CKM stages (*n* = 16,466).

CKM stage	*n* ^a^	OBS type	CVD mortality						All-cause mortality					
			Model 1			Model 2			Model 1			Model 2		
			HR	95% CI	*p*-value	HR	95% CI	*p*-value	HR	95% CI	*p*-value	HR	95% CI	*p*-value
Non-advanced	**13,387**	Total score	0.96	0.94, 0.98	**<0.001**	0.96	0.93, 0.99	**0.012**	0.97	0.96, 0.98	**<0.001**	0.97	0.95, 0.99	**<0.001**
		Diet score	0.97	0.94, 0.99	**0.003**	0.97	0.94, 1.00	**0.034**	0.97	0.96, 0.98	**<0.001**	0.97	0.96, 0.99	**0.006**
		Life score	0.87	0.79, 0.96	**0.005**	0.93	0.83, 1.03	0.200	0.90	0.86, 0.95	**<0.001**	0.92	0.87, 0.97	**0.003**
Advanced	**3,079**	Total score	0.97	0.96, 0.99	**<0.001**	0.97	0.94, 0.99	**0.014**	0.98	0.98, 0.99	**<0.001**	0.99	0.97, 1.00	0.094
		Diet score	0.97	0.96, 0.99	**<0.001**	0.98	0.95, 1.00	0.055	0.98	0.97, 0.99	**<0.001**	0.99	0.98, 1.01	0.200
		Life score	0.98	0.91, 1.06	0.700	0.89	0.81, 0.97	**0.010**	1.04	1.00, 1.09	0.076	0.96	0.91, 1.01	0.120

a Unweighted number of participants.

Model 1 Crude model.

Model 2 adjusted for age, gender, race, poverty income ratio (PIR), education level, marital status, log transformed values including white blood cell count, serum uric acid, and dietary intakes (energy, protein, carbohydrate, sodium and potassium).

OBS, oxidative balance score; CKM, Cardiovascular-Kidney-Metabolic Syndrome; CVD, Cardiovascular Disease; HR, hazard ratio; CI, confidential interval. Bold values represent statistical significance (*p* < 0.05).

In advanced CKM, total OBS was associated with reduced CVD mortality (HR 0.97, 95% CI 0.94–0.99, *p* = 0.014), but not all-cause mortality (*p* = 0.094). Life OBS was linked to lower CVD mortality (HR 0.89, 95% CI 0.81–0.97, *p* = 0.010) but not all-cause mortality. Diet OBS in advanced CKM was not significantly associated with either CVD or all-cause mortality.

We also examined the link between OBS and mortality across different CKM stages (Supplementary Table S5). Overall, the findings are mostly consistent with those from former analysis, particularly in the non-advanced CKM stages (Stage 0–2). In these stages, higher total OBS and diet OBS were typically associated with decreased all-cause mortality risk. However, the associations weakened or became non-significant in the advanced CKM stages (Stage 3–4), highlighting the potential diminishing effect of OBS on mortality as CKM progresses. No nonlinear association was observed between overall OBS and mortality across different CKM stages (Supplementary Figure S1).

### 3.4 Survival analysis

**Figure 4:**
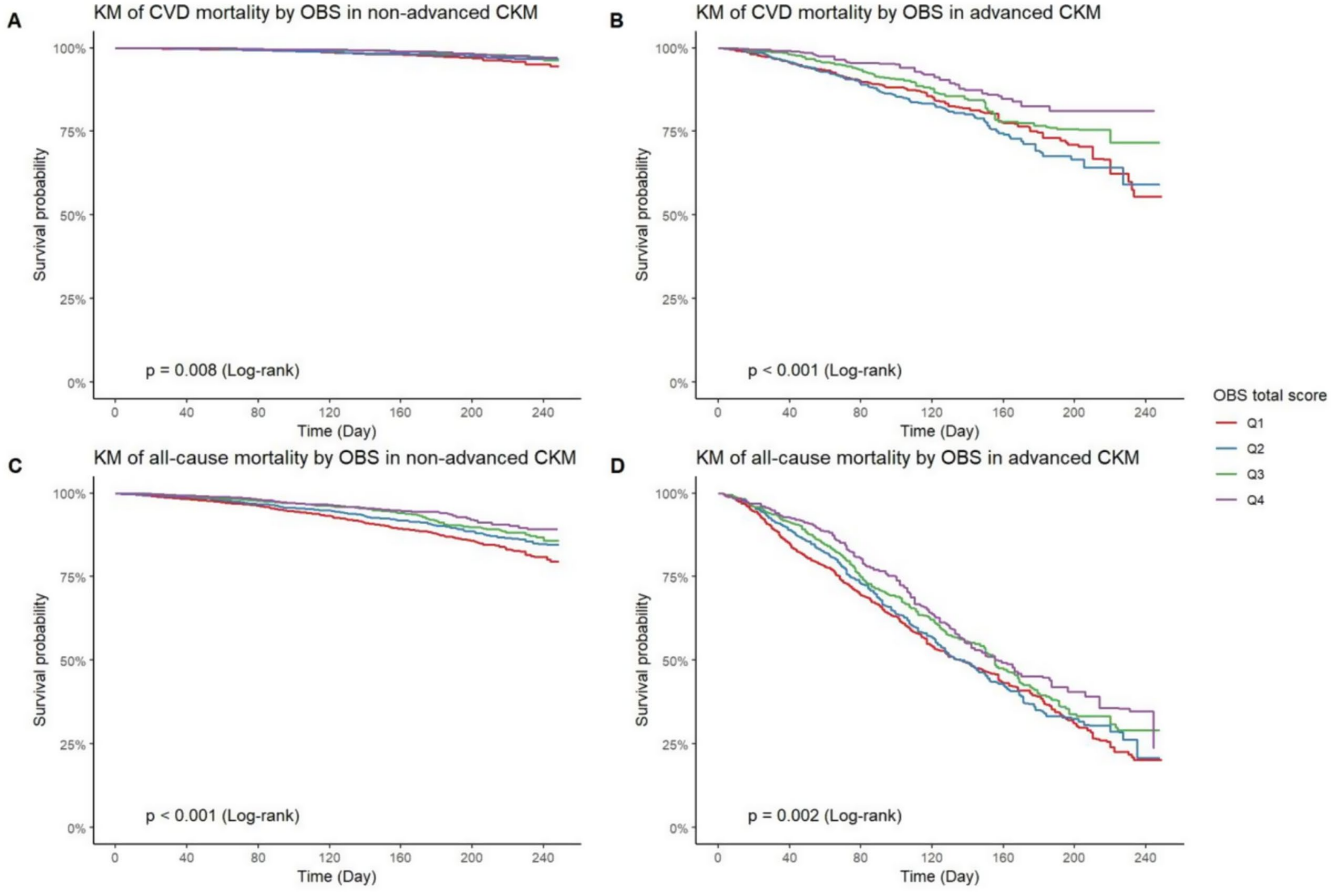
KM curves of OBS on CVD and all-cause mortality in binary CKM stages. KM curve of CVD mortality by OBS quartiles in non-advanced CKM **(A)** and advanced CKM **(B)**. KM curve of all-cause mortality by OBS quartiles in non-advanced CKM **(C)** and advanced CKM **(D)**. KM curve, Kaplan–Meier curve; OBS, oxidative balance score; CVD, cardiovascular diseases; CKM, Cardiovascular-Kidney-Metabolic syndrome.

As shown in Figure 4, Kaplan–Meier curves indicate significant differences in CVD and all-cause mortality across OBS quartiles among individuals with non-advanced CKM. Specifically, those in the highest OBS quartile (Q4) had lower CVD mortality (*p* = 0.008, Figure 4A) and all-cause mortality (*p* < 0.001, Figure 4C) compared to the lowest quartile (Q1). In advanced CKM, similar patterns were observed, with Q4 showing better survival for both CVD mortality (*p* < 0.001, Figure 4B) and all-cause mortality (*p* = 0.002, Figure 4D), though the differences were less pronounced than in the non-advanced group.

To account for competing events, Cumulative Incidence Function (CIF) and Fine–Gray test were employed. Among those with non-advanced CKM, higher OBS quartiles exhibit significantly lower cumulative incidence of CVD mortality (*p* < 0.001, Supplementary Figure S2A). In contrast, for advanced CKM (Supplementary Figure S2B), although there remains a discernible trend favoring higher OBS quartiles (lower cumulative incidence), the differences are not statistically significant (*p* = 0.067). Collectively, these results suggest that a higher OBS is associated with a lower risk of both CVD mortality and all-cause mortality, especially in individuals with non-advanced CKM.

### 3.5 Mediation analysis

**Figure 5:**
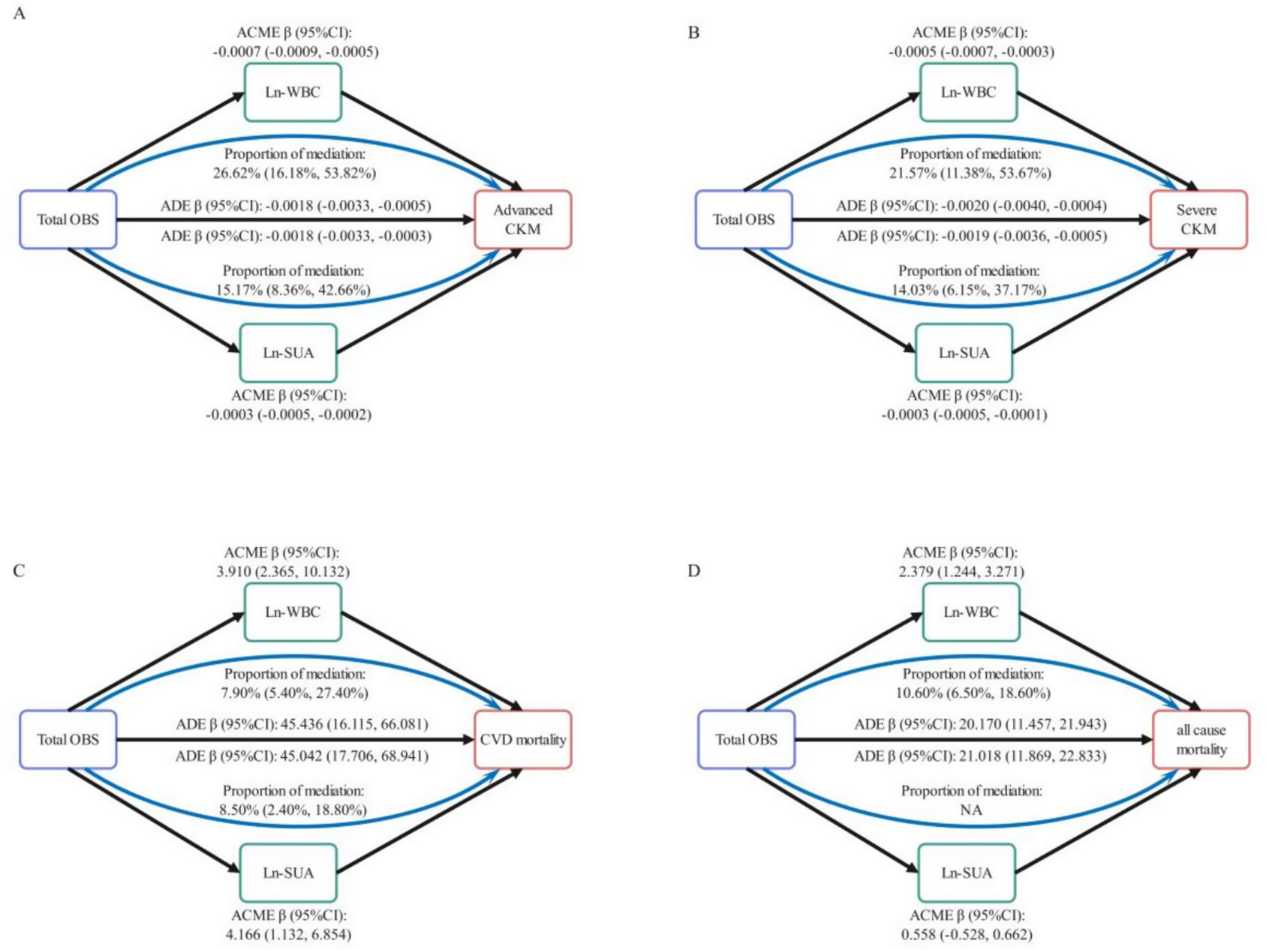
Mediation analysis in the association between total OBS and CKM Stages with CVD and all-cause mortality. Mediating role of Ln-WBC and Ln-SUA in the relationship of total OBS with advanced CKM **(A)** and sever CKM **(B)**. Mediating role of Ln-WBC and Ln-SUA in the relationship of total OBS with CVD mortality **(C)** and all-cause mortality **(D)** in non-advanced CKM stages. Ln, log transformed; WBC, white blood cells; SUA, serum uric acid; CVD, cardiovascular diseases; ACME, average causal mediation effect; ADE, average direct effect.

For advanced CKM (Figure 5A), the proportion of the total effect driven by Ln-WBC and Ln-SUA is statistically significant, accounting for 26.08% (95% CI: 16.18, 53.82%) and 15.17% (95% CI: 8.36, 42.66%), respectively. For severe CKM (Figure 5B), the proportion of the total effect accounted for by Ln-WBC and Ln-SUA is also statistically significant, accounting for 21.57% (95% CI: 11.38, 53.67%) and 14.03% (95% CI: 6.15, 37.17%), respectively. In participants with non-advanced CKM stages, for CVD mortality (Figure 5C), the proportion of the total effect contributed by Ln-WBC and Ln-SUA is statistically significant, accounting for 7.90% (95% CI: 5.40, 27.40%) and 8.50% (95% CI: 2.40, 18.80%), respectively. For all-cause mortality (Figure 5D), only Ln-WBC shows a statistically significant mediated proportion (10.60, 95% CI: 6.50, 18.60%), while the mediated proportion for Ln-SUA is not significant (*p* = 0.848).

## 4 Discussion

This research delivered robust insights into the links between OBS and CKM syndrome, as well as mortality outcomes across different CKM stages among a nationally representative cohort of U.S. individuals. Our findings indicated that higher OBS, reflecting greater antioxidant exposure, was tied to a lower likelihood of CKM progression and mortality, particularly in non-advanced CKM stages. These results highlighted the potential importance of maintaining a favorable oxidative balance through diet and lifestyle modifications to mitigate CKM-related health risks.

The negative correlation between OBS and CKM stages indicates that greater antioxidant exposure might offer protection against the progression of CKM syndrome. This finding aligns with mechanistic studies showing that antioxidants such as polyphenols, flavonoids, and Vitamins C and E can directly neutralize reactive oxygen species (ROS), thereby reducing oxidative impairment of lipids, proteins, and DNA (24, 25). Curcumin, derived from *Curcuma longa*, is known for its potent antioxidant properties, which are even stronger than those of vitamin C (26). However, its clinical application has been limited due to its poor water solubility and low bioavailability. Recent advancements in formulation strategies have shown that incorporating curcumin into submicron lipid carriers can significantly enhance its bioaccessibility and cellular uptake (27). These formulations not only enhance the delivery of curcumin to target tissues but also modulate key antioxidant genes, thereby providing robust protection against oxidative stress.

Oxidative stress disrupts cellular homeostasis through several pathways: metabolically, it impairs insulin signaling and reduces glucose transporter 4 (GLUT4) activity, exacerbating hyperglycemia and insulin resistance, but can be mitigated by antioxidants like *α*-lipoic acid (28). In the cardiovascular system, ROS promote endothelial dysfunction by reducing nitric oxide availability and activating pro-inflammatory pathways, while dietary flavonoids such as quercetin can improve endothelial function (29). In the kidneys, oxidative stress accelerates glomerulosclerosis and tubular fibrosis, but selenium can protect renal cells by scavenging peroxynitrite (30).

The protective effects of OBS vary by CKM stage. In stage 1 (metabolic risk factors), diet and lifestyle interventions are most effective, as oxidative damage is reversible. For example, a Mediterranean diet can reduce hepatic fat and improve lipid profiles in prediabetic individuals (31). In stages 3–4 (established CVD/CKD), antioxidants may have limited effects due to irreversible organ damage, but combining them with other therapies (e.g., statins or SGLT2 inhibitors) can enhance benefits (32). Certain antioxidants may exhibit U-shaped dose–response relationships. High-dose vitamin E (>400 IU/day) has been linked to increased hemorrhagic stroke risk and pro-oxidant effects in smokers, while synthetic beta-carotene increased lung cancer incidence in smokers, emphasizing the need for balanced, food-based intake and context-specific recommendations (33).

The marked association between higher OBS and diminished mortality risk, particularly in non-advanced stages of CKM syndrome, highlights the potential protective role of antioxidant exposure against adverse health outcomes. This finding aligns with prior research indicating that higher dietary antioxidant intake is associated with lower mortality from CVD and other chronic diseases. For example, a study using NHANES data with 10,591 patients with diabetes and prediabetes showed that each one-unit increase in OBS was linked to a 1.8% reduction in all-cause mortality risk and a 4% reduction in cardiovascular mortality risk (34). Another study also found that higher dietary antioxidant intake significantly reduces all-cause mortality in patients with depression (35). Moreover, the intake of plant-derived nitrates, which can be converted into nitric oxide in the body, has also been associated with reduced overall and cardiovascular mortality due to their antioxidant and cardiovascular protective effects (36). In non-advanced CKM stages, early intervention to improve oxidative balance may be particularly effective, as oxidative stress-induced damage is often reversible at this stage. The Mediterranean diet, rich in antioxidants, consistently reduces the risk of cardiovascular diseases and all-cause mortality. This diet, featuring plant-based foods, olive oil, and moderate fish and poultry intake, lowers oxidative stress and improves cardiovascular health. Adherence to it reduces serum lipids, blood pressure, and inflammation, lowering the incidence of cardiovascular events. It is also linked to a reduced risk of chronic diseases like cancer, diabetes, and neurodegenerative disorders (2). Furthermore, personalized nutritional interventions customized to an individual’s genetic and metabolic profiles may further enhance these protective effects (37).

The mediation analysis revealed that inflammatory markers, specifically Ln-WBC and Ln-SUA, significantly mediate the association between OBS and CKM outcomes. These findings epidemiologically validate inflammation as a central mechanism by which oxidative imbalance drives multiorgan metabolic dysfunction. Elevated WBC reflects chronic low-grade inflammation, while uric acid—acting as both an endogenous antioxidant and pro-inflammatory mediator—may form a pathogenic feedback loop in CKM progression (38, 39). Oxidative stress has been shown to trigger the activation of key signaling pathways, specifically NF-κB and Mitogen-Activated Protein Kinase (MAPK). Initiating these pathways sparks a chain reaction that eventually results in the assembly of the NLRP3 inflammasome. This intricate complex is vital for the immune response, as it enables the release of key pro-inflammatory cytokines like IL-1β and IL-18. These molecules are essential drivers of inflammation, orchestrating the body’s inflammatory cascade (40). These processes contribute to endothelial dysfunction, insulin resistance, and renal fibrosis. Notably, preclinical studies have demonstrated that antioxidant supplementation, such as with N-acetylcysteine or coenzyme Q10, can reduce inflammatory markers and reverse cardiorenal damage in high-fat diet models, providing causal evidence for the “oxidation-inflammation-organ injury” axis (41).

## 5 Strengths of this study

The strengths of this study include its large sample size and national representation, providing robust and generalizable findings. The comprehensive calculation of the OBS based on multiple dietary and lifestyle factors offers a holistic assessment of oxidative status. The study employs a wide range of advanced statistical techniques, including multinomial regression, Cox models, and mediation analysis, to thoroughly examine the relationships between OBS, CKM stages, and mortality outcomes. The use of well-defined CKM staging criteria ensures accurate participant categorization, while mediation analysis provides insights into potential biological mechanisms. Kaplan–Meier curves and survival analysis further highlight the protective effects of higher OBS values on mortality risks. Overall, this study makes a novel contribution to public health by emphasizing the importance of maintaining a favorable oxidative balance to mitigate CKM-related health risks.

## 6 Limitations of this study

Despite the notable strengths of our study, including its substantial sample size and the use of nationally representative data. It is important to acknowledge that these advantages do not preclude the presence of certain limitations that could influence the interpretation of our findings. Initially, it is important to recognize that the OBS is derived from a composite of self-reported dietary intake and lifestyle factors. These self-reported measures are inherently susceptible to measurement error, which may introduce some degree of uncertainty into our analysis.

Moreover, the cross-sectional design of the NHANES data poses a significant limitation in our ability to infer causality. While we can identify associations between variables, establishing a clear cause-and-effect relationship is challenging without longitudinal data. Future research should prioritize longitudinal studies to elucidate the temporal dynamics between the OBS and CKM outcomes, thereby providing more definitive insights into these relationships. Additionally, although we adjusted for a broad range of covariates, the potential for residual confounding cannot be entirely ruled out. There may be unmeasured or unaccounted-for variables that could influence the observed associations, and this residual confounding could potentially affect the robustness of our findings. Lastly, the generalizability of our findings to other populations may be constrained. Variations in dietary patterns and lifestyle factors across different regions and cultures could mean that the specific relationships identified in our study may not be universally applicable. Further research in diverse populations is needed to affirm and enrich our results.

## 7 Conclusion

To summarize, our study demonstrated a significant link between higher OBS and reduced cardiovascular and all-cause mortality, indicating that improving oxidative balance may be crucial for mitigating CKM syndrome risks. These findings emphasize the importance of maintaining a favorable oxidative balance for cardiovascular health. Future research should focus on validating these results in larger, more diverse cohorts to ensure generalizability. Additionally, clinical validation studies and experimental research are needed to elucidate the mechanisms underlying the impact of oxidative balance on CKM outcomes and to explore its therapeutic potential. Our study lays the groundwork for further investigation into oxidative balance as a target for improving CKM syndrome outcomes.

## Data Availability

The datasets presented in this study can be found in online repositories. The names of the repository/repositories and accession number(s) can be found in the article/Supplementary material.

## Glossary

AHA: American Heart Association
BMI: body mass index
CI: confidence intervals
CIF: Cumulative Incidence Function
CDC: Centers for Disease Control and Prevention
CKD: chronic kidney disease
CKM: Cardiovascular-Kidney-Metabolic
CVD: cardiovascular disease
GLUT4: glucose transporter 4
HR: hazard ratio
IQR: interquartile ranges
KDIGO: Kidney Disease Improving Global Outcomes
MET: metabolic equivalent of task
MAPK: Mitogen-Activated Protein Kinase
NDI: National Death Index
NHANES: National Health and Nutrition Examination Survey
OBS: Oxidative Balance Score
OR: Odds ratios
PA: physical activity
PIR: poverty income ratio
ROS: reactive oxygen species
RCS: restricted cubic spline
SD: standard deviations
T2DM: type 2 diabetes
