# Supplementary material for: The role of oxidative balance score in Cardiovascular-Kidney-Metabolic syndrome progression and mortality: insights from NHANES 1999–2020
Source: Front Nutr. 2025 Jul 1;12:1597693. doi: 10.3389/fnut.2025.1597693 (PMC12259437; doi:10.3389/fnut.2025.1597693)
Supplement: Supplementary file 1 [file Image_1.pdf]

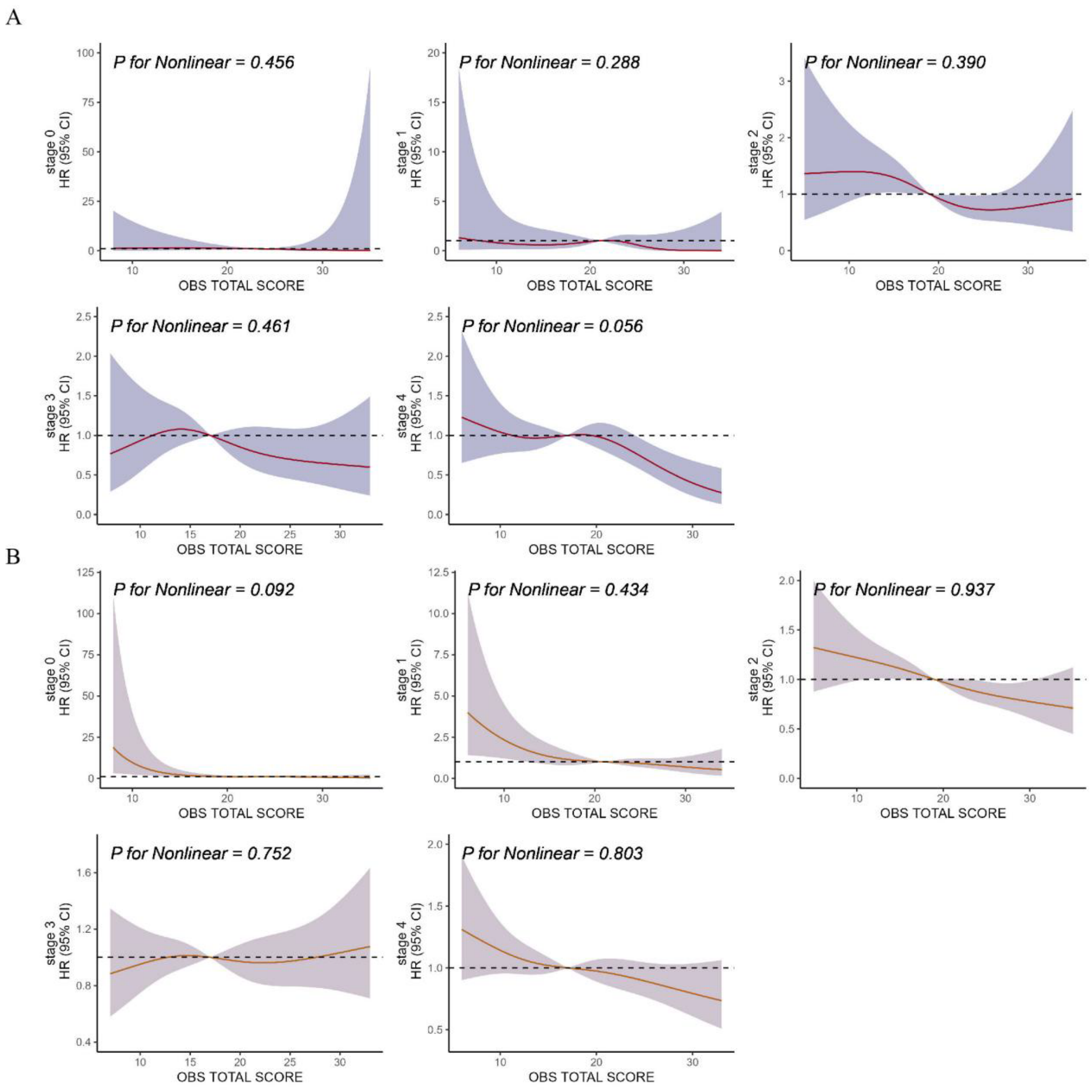

Figure S1. Associations of total oxidative balance score (OBS) with CVD mortality (A) and all-cause mortality (B) in each CKM stages. The horizontal dashed line represents to the reference hazard ratio of 1.0. All of the models were adjusted for age, gender, race, poverty-income ratio (PIR), education level, marital status, log transformed value of white blood cell count and serum uric acid, as well as dietary intakes of energy, protein, carbohydrate, sodium, and potassium.

Abbreviations: HR: hazard ratio; CI: confidence interval; CKM, Cardiovascular-Kidney-Metabolic syndrome

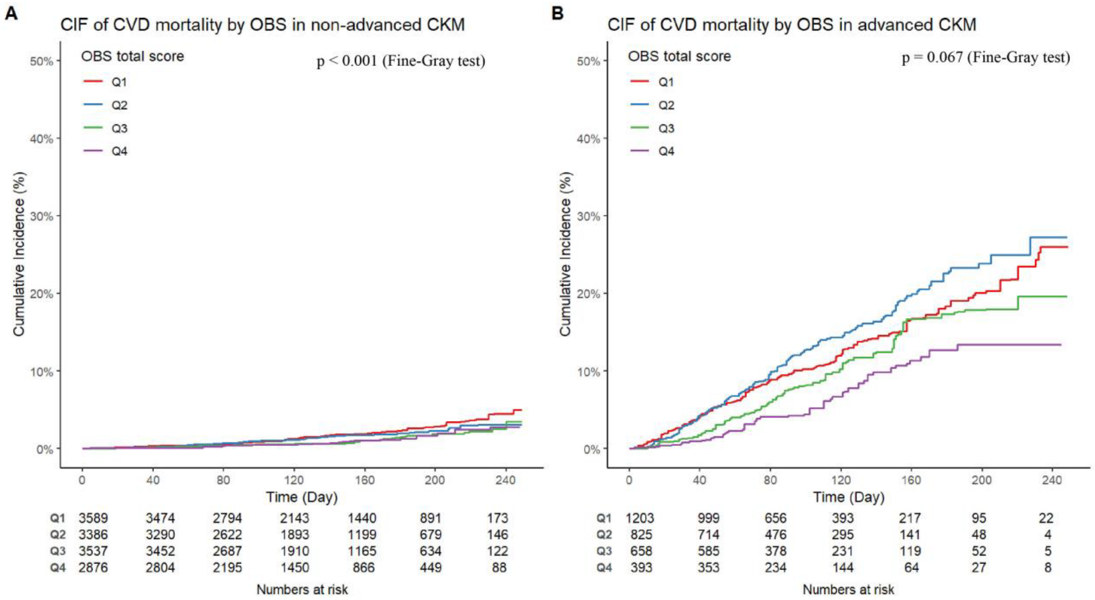

Figure S2. Cumulative Incidence Function (CIF) of cardiovascular disease (CVD) mortality by total OBS in non-advanced(A) and advanced(B) CKM stages.

Abbreviations: CKM, Cardiovascular-Kidney-Metabolic syndrome; OBS, oxidative balance score
