# Supplementary material for: The role of oxidative balance score in Cardiovascular-Kidney-Metabolic syndrome progression and mortality: insights from NHANES 1999–2020
Source: Front Nutr. 2025 Jul 1;12:1597693. doi: 10.3389/fnut.2025.1597693 (PMC12259437; doi:10.3389/fnut.2025.1597693)
Supplement: Supplementary file 2 [file Table_1.docx]

| **Table S1. Cardiovascular-Kidney-Metabolic Syndrome (CKM) definition ^[1]^** | | | | |  |  |  |
| --- | --- | --- | --- | --- | --- | --- | --- |
| **CKM Stage** | **Weight Criteria** | **Metabolic Criteria** | **Kidney Criteria** | **Cardiovascular Criteria** |  |  |  |
| Stage 0 | Normal BMI: <23 kg/m² (Asian)  Normal BMI: <25 kg/m² (Other) | Normal Waist Circumference: Women <80 cm (Asian), <88 cm (Other)  Men <90 cm (Asian), <102 cm (Other) | None | None |  |  |  |
| Stage 1 | Overweight BMI: ≥23 kg/m² (Asian) Overweight BMI: >25 kg/m² (Other) | 1. Overweight Waist Circumference:  Women ≥ 80 cm (Asian), ≥88 cm (Other)  Men ≥90 cm (Asian), ≥102 cm (Other)  2. or Prediabetes:   2.1. HbA1c 5.7%-6.5%   2.2. Fasting Blood Glucose 100-126 mg/dL | None | None |  |  |  |
| Stage 2 | None | 1. Elevated Triglycerides: ≥135 mg/dL  2. or Hypertension  3. or Diabetes  4. or Metabolic Syndrome (≥3 of the following):   4.1. Overweight Waist Circumference,   4.2. Low HDL Cholesterol (<40 mg/dL for men, <50 mg/dL for women),   4.3. Elevated Triglycerides ≥150 mg/dL, 4.4. Elevated Blood Pressure: elevated blood pressure   4.3.1 systolic blood pressure ≥130,   4.3.2 diastolic blood pressure ≥80 mmHg,   4.3.3 and/or use of blood pressure-lowering medications 5. Prediabetes | Moderate-to-High-Risk CKD (per KDIGO ^[2]^ criteria): CKD stages were identified based on GFR and urinary albumin-to creatinine ratio | None |  |  |  |
| Stage 3 | None | None | Very-High-Risk KDIGO CKD Stages | High Predicted 10-Year CVD Risk: ≥20% (using AHA PREVENT ^[3]^ equations) PREVENT was developed for variables with the following ranges:  1. total cholesterol 130-320 mg/dL,  2. HDL 20-100 mg/dL,  3. systolic blood pressure 90-200 mmHg,  4. and GFR 14-140 mL/min/1.73m². |  |  |  |
| Stage 4 | None | None | None | Established Cardiovascular Disease: Coronary Heart Disease, Angina, Heart Attack, Heart Failure, Stroke |  |  |  |
| Abbreviations: CKM, Cardiovascular-Kidney-Metabolic Syndrome; HDL: High-Density Lipoprotein; CKD: Chronic Kidney Disease; CVD: Cardiovascular Disease; ASCVD: Atherosclerotic Cardiovascular Disease | | | | |  |  |  |
| [1] Ndumele CE, Rangaswami J, Chow SL, et al. Cardiovascular-Kidney-Metabolic Health: A Presidential Advisory from the American Heart Association. Circulation. 2023; 148:16061635. | | | | |  |  |  |
| [2] Inker LA, Eneanya ND, Coresh J, et al. New Creatinine- and Cystatin C-Based Equations to Estimate GFR without Race. N Engl J Med. 2021; 385:1737-1749. | | | | |  |  |  |
| [3] Khan SS, Matsushita K, Sang Y, et al. Development and Validation of the American Heart Association Predicting Risk of Cardiovascular Disease EVENTs (PREVENT) Equations. Circulation. 2024; 149:430-449 | | | | |  |  |  |
|  |  |  |  |  |  |  |  |

| **Table S2. Oxidative balance score (OBS) criterion.** | | | | | | | |
| --- | --- | --- | --- | --- | --- | --- | --- |
| **OBS components** | **Property** | **Female** | | | **Male** | | |
|  |  | **0** | **1** | **2** | **0** | **1** | **2** |
| **Dietary OBS** |  |  |  |  |  |  |  |
| Dietary fiber (g/d) | A | ＜10.90 | 10.90- 16.80 | ≥16.80 | ＜13.15 | 13.15- 20.45 | ≥20.45 |
| Carotene (RE/d) 1999-2000 | A | ＜105.10 | 105.10- 371.55 | ≥371.55 | ＜109.29 | 109.29- 331.94 | ≥331.94 |
| Carotene (RE/d) 2001-2020 | A | ＜108.67 | 108.67- 392.89 | ≥392.89 | ＜104.89 | 104.89- 344.76 | ≥344.76 |
| Riboflavin (mg/d) | A | ＜1.43 | 1.43- 2.05 | ≥2.05 | ＜1.84 | 1.84- 2.68 | ≥2.68 |
| Niacin (mg/d) | A | ＜16.13 | 16.13- 23.02 | ≥23.02 | ＜22.99 | 22.99- 32.47 | ≥32.47 |
| Vitamin B6 (mg/d) | A | ＜1.25 | 1.25- 1.86 | ≥1.86 | ＜1.72 | 1.72- 2.54 | ≥2.54 |
| Total Folate (mcg/d) | A | ＜257.00 | 257.00- 386.50 | ≥386.50 | ＜330.00 | 330.00- 498.00 | ≥498.00 |
| Vitamin B12(mcg/d) | A | ＜2.54 | 2.54- 4.47 | ≥4.47 | ＜3.68 | 3.68- 6.34 | ≥6.34 |
| Vitamin C (mg/d) | A | ＜37.60 | 37.60- 86.60 | ≥86.60 | ＜39.75 | 39.75- 98.40 | ≥98.40 |
| Vitamin E (ATE) (mg/d) | A | ＜4.82 | 4.82- 7.84 | ≥7.84 | ＜6.01 | 6.01- 9.70 | ≥9.70 |
| Calcium (mg/d) | A | ＜600.50 | 600.50- 924.50 | ≥924.50 | ＜752.50 | 752.50- 1169.00 | ≥1169.00 |
| Magnesium (mg/d) | A | ＜205.50 | 205.50- 287.50 | ≥287.50 | ＜262.50 | 262.50- 369.00 | ≥369.00 |
| Zinc (mg/d) | A | ＜7.29 | 7.29- 10.47 | ≥10.47 | ＜10.20 | 10.20- 14.81 | ≥14.81 |
| Copper (mg/d) | A | ＜0.86 | 0.86- 1.22 | ≥1.22 | ＜1.08 | 1.08- 1.54 | ≥1.54 |
| Selenium (mcg/d) | A | ＜73.40 | 73.40- 105.45 | ≥105.45 | ＜102.75 | 102.75- 146.80 | ≥146.80 |
| Iron (mg/d) | P | ≥14.18 | 9.82- 14.18 | ＜9.82 | ≥18.89 | 13.04- 18.89 | ＜13.04 |
| Total fat (gm/d) | P | ≥77.83 | 53.52- 77.83 | ＜53.52 | ≥107.49 | 72.47- 107.49 | ＜72.47 |
|  |  |  |  |  |  |  |  |
| **Lifestyle OBS** |  |  |  |  |  |  |  |
| Alcohol consumption (g/d) | P | ≥15 | (0, 15) | non | ≥30 | (0, 30) | non |
| Serum cotinine (ng/mL) | P | ≥0.13 | 0.02- 0.13 | ＜0.02 | ≥2.46 | 0.03- 2.46 | ＜0.03 |
| Body mass index (Asian, kg/m2) | P | ≥30 | [23, 30) | ＜23 | ≥30 | [23, 30) | ＜23 |
| Body mass index (Other races, kg/m2) | P | ≥30 | [25, 30) | ＜25 | ≥30 | [25, 30) | ＜25 |
| Physical activity (MET-minutes/week) 1999-2006 | A | ＜339.15 | 339.15- 1898.40 | ≥1898.40 | ＜283.50 | 283.50- 1638.00 | ≥1638.00 |
| Physical activity (MET-minutes/week) 2007-2020 | A | ＜1440.00 | 1440.00- 9200.00 | ≥9200.00 | ＜2080.00 | 2080.00- 9720.00 | ≥9720.00 |
| Abbreviations: OBS, oxidative balance score; MET, Metabolic Equivalent of Task | | |  |  |  |  |  |

| **Table S3. Weighted number of participants of each CKM stage across NHANES cycles 1999-2000.** | | | | | | |
| --- | --- | --- | --- | --- | --- | --- |
| NHANES Cycles | Overall | CKM 0 | CKM 1 | CKM 2 | CKM 3 | CKM 4 |
| 1999-2000 | 14,168,601 | 1,920,885 (13.56%) | 2,054,626 (14.50%) | 8,265,534 (58.34%) | 551,650 (3.89%) | 1,375,906 (9.71%) |
| 2001-2002 | 19,303,868 | 2,693,202 (13.95%) | 3,172,788 (16.43%) | 10,868,269 (56.31%) | 721,248 (3.74%) | 1,848,362 (9.57%) |
| 2003-2004 | 16,874,583 | 2,238,019 (13.27%) | 2,667,017 (15.81%) | 9,550,256 (56.60%) | 532,757 (3.16%) | 1,886,534 (11.18%) |
| 2005-2006 | 16,907,433 | 2,156,181 (12.75%) | 3,097,791 (18.32%) | 9,350,989 (55.30%) | 680,849 (4.03%) | 1,621,623 (9.59%) |
| 2007-2008 | 16,927,983 | 1,900,223 (11.23%) | 3,636,488 (21.49%) | 9,127,001 (53.93%) | 617,385 (3.65%) | 1,646,886 (9.73%) |
| 2009-2010 | 17,522,261 | 2,107,266 (12.03%) | 4,186,100 (23.89%) | 8,994,126 (51.35%) | 660,586 (3.77%) | 1,574,184 (8.99%) |
| 2011-2012 | 18,011,426 | 1,750,190 (9.72%) | 3,831,759 (21.28%) | 10,032,429 (55.71%) | 586,627 (3.26%) | 1,810,421 (10.05%) |
| 2013-2014 | 17,747,055 | 1,748,007 (9.84%) | 4,128,106 (23.26%) | 9,605,780 (54.13%) | 575,054 (3.24%) | 1,690,108 (9.52%) |
| 2015-2016 | 17,811,137 | 1,573,134 (8.83%) | 4,312,278 (24.21%) | 9,544,758 (53.59%) | 572,398 (3.21%) | 1,808,569 (10.16%) |
| 2017-2020 | 29,901,542 | 2,867,934 (9.59%) | 7,171,258 (23.99%) | 15,699,037 (52.48%) | 955,222 (3.20%) | 3,208,092 (10.73%) |
| Abbreviations: CKM, Cardiovascular-Kidney-Metabolic Syndrome | | | | | | |

| **Table S4. Baseline characteristics of participants according to the OBS quartiles, NHANES 1999-2020.** | | | | | | |
| --- | --- | --- | --- | --- | --- | --- |
| **Characteristic** | **Overall,  n = 19,433  N=185,175,890^1^** | **Q1 [0,14) n = 5,742 (26%) N=48,352,374** | **Q2 [14,20) n = 4,980 (25%) N=46,579,321** | **Q3 [20,26) n = 4,918 (27%) N=49,463,463** | **Q4 ≥26 n = 3,793 (22%) N=40,780,733** | **p-value^2^** |
| **Age (years old)** | 47.00 (34.00, 60.00) | 47.00 (33.00, 61.00) | 47.00 (34.00, 61.00) | 47.00 (34.00, 60.00) | 46.00 (34.00, 59.00) | 0.107 |
| **Gender** |  |  |  |  |  | 0.149 |
| **Female** | 9,476 (48.18%) | 2,899 (48.74%) | 2,471 (49.47%) | 2,356 (47.92%) | 1,750 (46.38%) |  |
| **Male** | 9,957 (51.82%) | 2,843 (51.26%) | 2,509 (50.53%) | 2,562 (52.08%) | 2,043 (53.62%) |  |
| **Poverty income ratio (PIR)** |  |  |  |  |  | **<0.001** |
| **≤1.3** | 5,162 (18.76%) | 1,934 (26.15%) | 1,306 (18.90%) | 1,131 (15.50%) | 791 (13.79%) |  |
| **1.3-3.5** | 6,898 (34.08%) | 2,117 (37.01%) | 1,863 (36.04%) | 1,689 (32.79%) | 1,229 (29.94%) |  |
| **≤3.5** | 5,787 (40.63%) | 1,181 (29.62%) | 1,403 (38.27%) | 1,702 (45.20%) | 1,501 (50.86%) |  |
| **Race** |  |  |  |  |  | **<0.001** |
| **Mexican American** | 3,314 (7.74%) | 926 (7.22%) | 852 (7.48%) | 857 (8.00%) | 679 (8.35%) |  |
| **Non-Hispanic White** | 3,863 (10.74%) | 1,585 (16.39%) | 993 (11.17%) | 810 (8.60%) | 475 (6.14%) |  |
| **Non-Hispanic Black** | 8,994 (69.72%) | 2,394 (64.58%) | 2,281 (68.80%) | 2,346 (71.15%) | 1,973 (75.14%) |  |
| **Other Race** | 3,262 (11.79%) | 837 (11.81%) | 854 (12.55%) | 905 (12.25%) | 666 (10.37%) |  |
| **Education** |  |  |  |  |  | **<0.001** |
| **Below High School** | 2,122 (5.46%) | 869 (8.30%) | 574 (5.83%) | 424 (4.08%) | 255 (3.35%) |  |
| **High School** | 7,162 (34.70%) | 2,600 (45.50%) | 1,845 (35.85%) | 1,643 (31.27%) | 1,074 (24.77%) |  |
| **Higher Than High School** | 10,149 (59.83%) | 2,273 (46.20%) | 2,561 (58.32%) | 2,851 (64.65%) | 2,464 (71.88%) |  |
| **Marital status** |  |  |  |  |  | **<0.001** |
| **Never Married** | 3,206 (16.81%) | 1,015 (18.66%) | 772 (16.02%) | 787 (16.33%) | 632 (16.12%) |  |
| **Married/Living with Partner** | 11,987 (65.20%) | 3,254 (59.61%) | 3,111 (65.48%) | 3,121 (66.82%) | 2,501 (69.55%) |  |
| **Others** | 4,240 (17.99%) | 1,473 (21.73%) | 1,097 (18.51%) | 1,010 (16.85%) | 660 (14.33%) |  |
| **Energy intake (Kcal/d)** | 1,998.10 (1,527.00, 2,571.88) | 1,475.06 (1,129.50, 1,877.85) | 1,894.21 (1,507.25, 2,334.50) | 2,215.42 (1,790.00, 2,740.57) | 2,587.01 (2,042.21, 3,247.18) | **<0.001** |
| **Protein intake (g/d)** | 76.62 (57.24, 100.66) | 52.74 (40.04, 66.49) | 72.16 (57.29, 88.89) | 86.20 (69.33, 106.58) | 107.10 (84.50, 133.00) | **<0.001** |
| **Carbohydrate intake (g/d)** | 237.51 (178.19, 310.85) | 175.65 (129.99, 229.00) | 221.38 (174.46, 280.83) | 261.12 (206.22, 324.95) | 310.84 (245.53, 395.00) | **<0.001** |
| **Sodium intake (mg/d)** | 3,227.67 (2,401.00, 4,242.50) | 2,347.66 (1,750.48, 3,033.00) | 3,121.65 (2,428.79, 3,866.72) | 3,601.30 (2,791.37, 4,599.82) | 4,233.28 (3,285.49, 5,528.41) | **<0.001** |
| **Potassium intake (mg/d)** | 2,552.50 (1,919.51, 3,255.00) | 1,653.30 (1,288.47, 2,040.98) | 2,342.28 (1,969.00, 2,784.07) | 2,877.50 (2,421.63, 3,428.50) | 3,632.50 (3,025.77, 4,389.07) | **<0.001** |
| **White blood cells (10^9^/L)** | 6.40 (5.40, 7.80) | 6.80 (5.60, 8.20) | 6.50 (5.50, 7.90) | 6.40 (5.40, 7.70) | 6.10 (5.10, 7.40) | **<0.001** |
| **Serum uric acid (umol/L)** | 321.20 (267.70, 380.70) | 333.10 (273.60, 386.60) | 327.10 (273.60, 380.70) | 315.20 (261.70, 374.70) | 303.30 (255.80, 356.90) | **<0.001** |
| **CKM stages** |  |  |  |  |  | **<0.001** |
| **stage 0** | 1,676 (11.32%) | 311 (7.08%) | 358 (9.68%) | 474 (12.14%) | 533 (17.20%) |  |
| **stage 1** | 3,604 (20.66%) | 918 (18.74%) | 847 (18.23%) | 989 (22.74%) | 850 (23.18%) |  |
| **stage 2** | 10,537 (54.56%) | 3,064 (55.74%) | 2,826 (57.36%) | 2,692 (54.04%) | 1,955 (50.59%) |  |
| **stage 3** | 1,171 (3.49%) | 450 (4.85%) | 323 (3.90%) | 240 (2.82%) | 158 (2.19%) |  |
| **stage 4** | 2,445 (9.97%) | 999 (13.58%) | 626 (10.81%) | 523 (8.25%) | 297 (6.84%) |  |
| **Body mass index (kg/m2)** | 27.79 (24.10, 32.22) | 28.85 (25.07, 33.40) | 28.11 (24.62, 32.59) | 27.69 (24.00, 32.07) | 26.35 (23.18, 30.30) | **<0.001** |
| **Waist (cm)** | 97.30 (87.00, 108.40) | 99.54 (89.00, 111.10) | 98.50 (88.30, 109.00) | 97.00 (86.80, 107.80) | 93.60 (83.94, 105.10) | **<0.001** |
| **Glucose (mg/dL)** | 99.00 (92.00, 107.50) | 99.70 (92.00, 109.00) | 99.00 (92.58, 108.80) | 99.00 (92.00, 107.00) | 97.10 (91.00, 105.00) | **<0.001** |
| **Glycohemoglobin (%)** | 5.40 (5.10, 5.70) | 5.40 (5.20, 5.80) | 5.40 (5.20, 5.70) | 5.40 (5.20, 5.70) | 5.40 (5.10, 5.60) | **<0.001** |
| **Triglycerides(mg/dL)** | 105.00 (72.00, 155.00) | 110.00 (77.00, 161.00) | 109.00 (75.00, 158.00) | 102.00 (71.00, 152.00) | 96.00 (65.00, 143.00) | **<0.001** |
| **HDL (mg/dL)** | 51.00 (42.00, 62.00) | 48.00 (41.00, 59.00) | 50.00 (42.00, 62.00) | 52.00 (42.00, 62.86) | 54.00 (44.00, 66.00) | **<0.001** |
| **Hypertension** | 10,562 (49.35%) | 3,475 (54.46%) | 2,803 (52.71%) | 2,537 (46.73%) | 1,747 (42.62%) | **<0.001** |
| **Metabolic syndromes** | 8,010 (38.18%) | 2,642 (43.05%) | 2,160 (40.99%) | 1,973 (37.16%) | 1,235 (30.43%) | **<0.001** |
| **Pre-Diabetes** | 9,570 (46.24%) | 2,895 (47.28%) | 2,506 (47.23%) | 2,441 (47.31%) | 1,728 (42.57%) | **<0.001** |
| **Glycemic Status** |  |  |  |  |  | **<0.001** |
| **Diabetes** | 3,706 (14.42%) | 1,325 (17.99%) | 1,010 (15.32%) | 843 (12.93%) | 528 (10.95%) |  |
| **Impaired Fasting Glucose (IFG)** | 1,862 (9.24%) | 586 (9.45%) | 474 (9.67%) | 502 (10.09%) | 300 (7.46%) |  |
| **Impaired Glucose Tolerance (IGT)** | 1,132 (5.25%) | 299 (4.47%) | 288 (5.36%) | 317 (5.46%) | 228 (5.81%) |  |
| **Normoglycemia** | 12,731 (71.09%) | 3,531 (68.09%) | 3,208 (69.65%) | 3,255 (71.52%) | 2,737 (75.78%) |  |
| **CKD Prognosis** | 3,338 (12.83%) | 1,287 (17.37%) | 869 (13.20%) | 706 (10.38%) | 476 (9.98%) | **<0.001** |
| **High CVD 10 years risk** | 3.57 (1.32, 9.09) | 4.50 (1.68, 11.97) | 3.86 (1.37, 9.92) | 3.33 (1.23, 8.39) | 2.73 (1.04, 6.78) | **<0.001** |
| **ASCVD** | 2,050 (8.48%) | 846 (11.62%) | 524 (9.18%) | 437 (7.03%) | 243 (5.72%) | **<0.001** |
| **Congestive Heart Failure** | 639 (2.43%) | 280 (3.57%) | 167 (2.70%) | 123 (1.94%) | 69 (1.35%) | **<0.001** |
| **NHANES cycles** |  |  |  |  |  | **<0.001** |
| **1999-2000** | 1,518 (7.65%) | 584 (10.04%) | 392 (7.89%) | 320 (6.70%) | 222 (5.70%) |  |
| **2001-2002** | 1,977 (10.42%) | 698 (13.61%) | 501 (10.26%) | 469 (9.82%) | 309 (7.57%) |  |
| **2003-2004** | 1,654 (9.11%) | 513 (9.14%) | 455 (9.65%) | 386 (8.91%) | 300 (8.71%) |  |
| **2005-2006** | 1,594 (9.13%) | 449 (8.59%) | 434 (9.50%) | 398 (8.98%) | 313 (9.53%) |  |
| **2007-2008** | 1,944 (9.14%) | 586 (8.79%) | 491 (8.79%) | 485 (8.77%) | 382 (10.40%) |  |
| **2009-2010** | 2,189 (9.46%) | 541 (7.60%) | 554 (9.35%) | 611 (9.97%) | 483 (11.19%) |  |
| **2011-2012** | 1,911 (9.73%) | 464 (7.71%) | 488 (9.27%) | 503 (9.80%) | 456 (12.54%) |  |
| **2013-2014** | 1,926 (9.58%) | 457 (8.13%) | 469 (9.12%) | 560 (10.72%) | 440 (10.46%) |  |
| **2015-2016** | 1,767 (9.62%) | 505 (9.35%) | 430 (9.49%) | 465 (9.34%) | 367 (10.41%) |  |
| **2017-2020** | 2,953 (16.15%) | 945 (17.04%) | 766 (16.67%) | 721 (16.97%) | 521 (13.48%) |  |
| ^1^Median (IQR) for skewed distribution; n (unweighted) (weighted percentage%); N (weighted) | | | | | | |
| ^2^Wilcoxon rank-sum test for complex survey samples; chi-squared test with Rao & Scott's second-order correction | | | | | | |
| Abbreviations: OBS, oxidative balance score; CKM, Cardiovascular-Kidney-Metabolic Syndrome; HDL: High-Density Lipoprotein; CKD: Chronic Kidney Disease; CVD: Cardiovascular Disease; ASCVD: Atherosclerotic Cardiovascular Disease | | | | | | |

| **Table S5. Cox regression of the relationship between OBS and mortality in each CKM stage. (n= 16,466)** | | | | | | | | | | | | | | |
| --- | --- | --- | --- | --- | --- | --- | --- | --- | --- | --- | --- | --- | --- | --- |
|  |  |  | **CVD mortality** | | | | | | **All-cause mortality** | | | | | |
|  |  |  | **Model 1** | | | **Model 2** | | | **Model 1** | | | **Model 2** | | |
| **CKM Stage** | **n^1^** | **OBS types** | **HR** | **95% CI** | **p-value** | **HR** | **95% CI** | **p-value** | **HR** | **95% CI** | **p-value** | **HR** | **95% CI** | **p-value** |
| **0** | **1,475** | **Total score** | 1.05 | 0.93, 1.18 | 0.400 | 0.80 | 0.74, 0.86 | **<0.001** | 0.95 | 0.90, 1.00 | 0.070 | 0.90 | 0.82, 0.99 | **0.023** |
|  |  | **Diet score** | 1.05 | 0.94, 1.18 | 0.400 | 0.76 | 0.71, 0.82 | **<0.001** | 0.95 | 0.90, 1.00 | **0.049** | 0.89 | 0.82, 0.97 | **0.006** |
|  |  | **Life score** | 1.00 | 0.55, 1.83 | 0.910 | 0.93 | 0.27, 3.18 | 0.900 | 0.98 | 0.72, 1.34 | 0.90 | 0.93 | 0.56, 1.55 | 0.800 |
| **1** | **3,004** | **Total score** | 0.95 | 0.90, 1.00 | **0.043** | 0.94 | 0.86, 1.03 | 0.200 | 0.96 | 0.93, 0.99 | **0.009** | 0.94 | 0.90, 0.99 | **0.019** |
|  |  | **Diet score** | 0.95 | 0.89, 1.00 | 0.063 | 0.93 | 0.82, 1.06 | 0.300 | 0.96 | 0.92, 0.99 | **0.011** | 0.95 | 0.90, 1.00 | 0.050 |
|  |  | **Life score** | 0.88 | 0.69, 1.13 | 0.300 | 0.97 | 0.68, 1.40 | 0.900 | 0.98 | 0.85, 1.12 | 0.700 | 0.88 | 0.76, 1.02 | 0.083 |
| **2** | **8,909** | **Total score** | 0.97 | 0.95, 0.99 | **0.009** | 0.96 | 0.93, 1.00 | **0.030** | 0.98 | 0.97, 0.98 | **<0.001** | 0.98 | 0.96, 1.00 | **0.015** |
|  |  | **Diet score** | 0.97 | 0.95, 0.99 | **0.013** | 0.97 | 0.93, 1.00 | 0.053 | 0.98 | 0.97, 0.99 | **<0.001** | 0.98 | 0.96, 1.00 | 0.064 |
|  |  | **Life score** | 0.94 | 0.84, 1.05 | 0.300 | 0.94 | 0.84, 1.06 | 0.300 | 0.96 | 0.91, 1.01 | 0.140 | 0.93 | 0.88, 0.98 | **0.010** |
| **3** | **1,022** | **Total score** | 1.00 | 0.98, 1.03 | 0.900 | 0.97 | 0.92, 1.03 | 0.300 | 1.00 | 0.98, 1.01 | 0.400 | 1.00 | 0.97, 1.03 | 0.900 |
|  |  | **Diet score** | 1.00 | 0.98, 1.03 | 0.700 | 0.99 | 0.93, 1.04 | 0.700 | 0.99 | 0.98, 1.01 | 0.300 | 1.00 | 0.97, 1.03 | 0.900 |
|  |  | **Life score** | 0.95 | 0.85, 1.08 | 0.400 | 0.86 | 0.74, 1.00 | 0.054 | 1.05 | 0.98, 1.13 | 0.150 | 1.03 | 0.95, 1.11 | 0.500 |
| **4** | **2,057** | **Total score** | 0.97 | 0.95, 0.98 | **<0.001** | 0.96 | 0.93, 0.99 | **0.018** | 0.98 | 0.97, 0.99 | **<0.001** | 0.98 | 0.96, 1.00 | 0.088 |
|  |  | **Diet score** | 0.96 | 0.95, 0.98 | **<0.001** | 0.97 | 0.94, 1.00 | **0.026** | 0.98 | 0.97, 0.99 | **<0.001** | 0.99 | 0.97, 1.01 | 0.200 |
|  |  | **Life score** | 0.98 | 0.89, 1.09 | 0.800 | 0.90 | 0.78, 1.03 | 0.120 | 0.99 | 0.94, 1.05 | 0.800 | 0.93 | 0.87, 0.99 | **0.023** |
| **^1^** unweighted number of participants | | | | | | | | | | | | | | |
| Model 1 Crude model | | | | | | | | | | | | | | |
| Model 2 adjusted for age, gender, race, poverty income ratio (PIR), education level, marital status, log transformed values including white blood cell count, serum uric acid, and dietary intakes (energy, protein, carbohydrate, sodium and potassium) | | | | | | | | | | | | | | |
| Abbreviations: OBS, oxidative balance score; CKM, Cardiovascular-Kidney-Metabolic Syndrome; CVD: Cardiovascular Disease; HR, hazard ratio; CI, confidential interval | | | | | | | | | | | | | | |
